# Multireference Modeling Reveals the Origins of L‑Edge X‑ray Absorption Features in Photoredox-Active Nickel Complexes

**DOI:** 10.1021/acs.jpcc.6c00223

**Published:** 2026-02-11

**Authors:** Olivia Ho, Shawna Lin, Thais R. Scott

**Affiliations:** Department of Chemistry, 2050 Bowdoin College, Brunswick, Maine 04011, United States

## Abstract

Nickel photoredox catalysts exhibit an unusual shoulder feature in the L_3_-edge X-ray absorption spectra, which has been attributed to multiconfigurational ground-state character. We investigate this hypothesis with multireference methods by comparing active spaces with primarily d-orbital character (d–d) and active spaces that replace a second shell d-orbital with a π* orbital on the bipyridine ligand to allow for metal–ligand charge transfer (MLCT). Through the d–d active space calculations, we find that the splitting between the main peak and the shoulder is a result of two spin–orbit states dominated by spin-free states with different spin multiplicities. MLCT active spaces better capture experimental peak-height ratios, supporting previous assignments of trends in the shoulder intensity. Overall, our analysis suggests that the shoulder arises from 2p to 3d transitions modulated by nearby π* orbitals rather than direct core 2p to π* excitations. These results demonstrate how active space composition and spin multiplicity impact L_3_-edge spectral features in these multiconfigurational transition-metal complexes.

## Graphical abstract

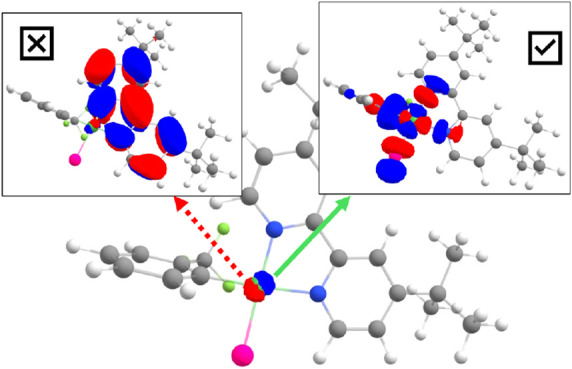

## Introduction

Nickel-based photoredox catalysts are powerful activation platforms for forging carbon–carbon and carbon–heteroatom bonds under mild conditions.^1,2^ Widespread adoption of this approach has prompted further investigation of the fundamental electronic structure of both the ground and excited states responsible for reactivity.^3–9^ Covalent mixing between d-orbitals on the nickel and unoccupied π*-orbitals on the bipyridine ligand has been suggested to play a key role in reactivity,^10^ and it has been previously reported that X-ray absorption spectra (XAS) support a multiconfigurational (MC) description of the ground state.^11^ Preliminary semiempirical analysis by Nelson et al., performed using charge-transfer multiplet (CTM) simulations to model X-ray absorption spectroscopy, suggested that the excited states responsible for the shoulder had metal–ligand charge-transfer character. Multireference computational approaches are an ideal tool for providing more detailed interpretations of the final states contributing to the core-level spectroscopic signatures in systems with MC character.^12,13^ Here, we identify the electronic excitations underlying the unexpected Ni L_3_-edge features and demonstrate the effect of ligand substitution.

In XAS, high-energy core-excited states are used to probe properties of unoccupied or partially occupied valence orbitals. Four nickel 2,2′-bipyridine aryl chloride (Ni­(bpy)­ArCl) complexes, shown in Figure 1a, were characterized through K-edge and L_2,3_-edge XAS in the study by Nelson and co-workers.^11^ In this work, we analyze these compounds with multireference methods to refine their interpretations. These square-planar Ni^II^ complexes have d^8^ singlet ground states, as established through structural and spectroscopic characterization.^4,14^ The four compounds in this series were selected because of the variation in the electron-donating groups on the bpy and aryl ligands. The *tert*-butyl substituent in **1B** and **5B** is expected to destabilize the π* manifold relative to the Ni d-orbitals, whereas the methyl acetate group in **1D** and **5D** likely has the opposite effect. In **1D** and **1B**, the electron-donating alkyl group on the aryl ligand enhances the σ-donation to the metal, destabilizing the Ni d-manifold. In contrast, **5D** and **5B** feature an electron-withdrawing trifluoromethyl group, which stabilizes the d-manifold.

**Figure 1:**
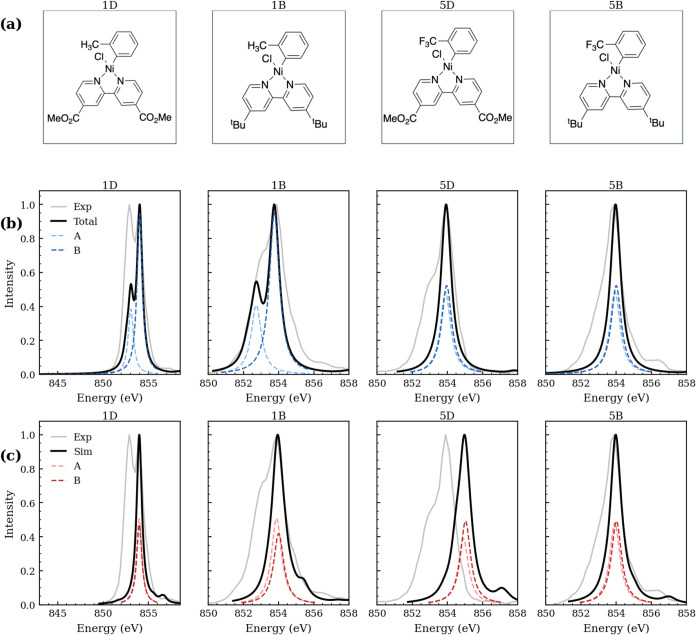
(a) Molecular structures of the compounds studied. Labels denote substituents: 1 vs 5 indicate the aryl substituent (1 = CH_3_, 5 = CF_3_) and B vs D indicate the bipyridine substituent (B = *tert*-butyl, D = methyl acetate). Rows (b) and (c) show the calculated Ni L_3_-edge spectra compared to the experiment: (b) d–d active-space results and (c) metal–ligand charge transfer (MLCT) active-space results. Each panel shows the experimental spectrum (gray), the full simulated spectrum (black), and the two components that contribute to the simulated spectra: peak A (light) and peak B (dark color). In (b), peaks A/B are light/dark blue; in (c), peaks A/B are light/dark red. Experimental and simulated spectra are normalized to unit peak height. Simulated spectra are shifted by −3.7 eV to align with the experiment, and Lorentzian broadened by 0.7 eV. Experimental data reproduced from ref 11.

The pre-edge features in the Ni K-edge for complex **1D** exhibit a pronounced blue shift.^11^ The intensity of those features was approximately an order of magnitude greater than that observed for previously reported square-planar Ni^II^ complexes.^15^ These spectral characteristics are attributed to increased 3d/4p orbital mixing and were corroborated by time-dependent density functional theory (TD–DFT) calculations.^11^ Given that these effects can be replicated with a single-reference approach such as TD-DFT, we do not investigate them further. We focus our efforts to explain the unusual features of the L_3_-edge.

For each complex, the L_3_-edge region displayed either a dual-peak structure or a clear shoulder, a rare feature in low-spin Ni^II^ complexes. In this work, we label the peak centered at 852.9 eV as peak A and the peak centered at 853.8 eV as peak B. This behavior was attributed to the MC character of the ground state, specifically, variations in orbital covalency, with differential contributions of metal and ligand character in the valence molecular orbitals.^11^ A correlation was observed between the percentage of MC character in the ground state in previous multireference calculations^16^ and the height of the peak/shoulder at 852.9 eV (peak A). This prompted the assignment of peak A to transitions between the core 2p orbitals and the π* orbitals located on the bipyridine ligand.^11^ Satellite features with lower intensity were also observed and separated by about 2 eV from the L_3_ peaks. The L_3_-edge absorption profiles of these nickel complexes exhibit spectral characteristics that are similar to those observed for excited state of monatomic Ni^+^ cations in the 3d^8^4s^1 4^F_9/2_ electron configuration and [NiF]^+^.^17^ This is noteworthy, given that the ground-state electronic configurations of these square-planar Ni^II^ complexes are expected to be diamagnetic d^8^ singlets. In another study, time-resolved Ni L- and K-edge XAS identified a metal-centered triplet excited state in these compounds.^18^ To assess the impact of ground-state MC character on spectroscopic features, we apply multireference electronic structure methods.

Our approach involves using complete active space self-consistent field theory (CASSCF) to target the spectral response to different types of excitations by varying the active space composition. We then employ a restricted active space approach with configuration interaction (RASCI)^19^ with the high-energy excitations (HEXS) method^20^ to target the core-excited states. Multiconfigurational pair density functional theory (MC-PDFT) was used on-top of the RASCI energies to incorporate additional electron correlation. The MC-PDFT method has been shown to have mean unsigned errors similar to complete active space with second-order perturbation theory (CASPT2) for core-excitations at a significantly reduced cost.^21^ The spin-free states are mixed with the RAS state interaction (RASSI) method to form spin–orbit states.^22,23^ Our analysis hinges on the choice of active space, which dictates the possible transitions that can contribute to the spin–orbit states.

The choice of active space must be made carefully, as an active space that is too small may exclude fundamental transitions that are key to spectral signatures and an active space that is too large would have unnecessary computational costs and little improved accuracy. The majority of multireference studies involving these compounds use an active space of 10 electrons and 9 orbitals, which incorporates d_*xy*_, d_*yz*_, d_*xz*_, d_*z* ^2^_, d_*x* ^2^–*y* ^2^_/σ (between the aryl group and the nickel), d_*x* ^2^–*y* ^2^_/σ* (between the aryl group and the nickel), and 3 π* orbitals on the on the bipyridine ligand.^10,16,24^ In the closed shell electron configuration the four d-orbitals and the Ni-aryl σ/d_*x* ^2^–*y* ^2^_ orbital are doubly occupied and the Ni-aryl σ*/d_*x* ^2^–*y* ^2^_ and bipyridine π* orbitals are unoccupied. Larger active space sizes with different compositions have also been investigated, like CAS­(12,12)^25^ and CAS­(12,16)^9^ to include orbitals localized on the halide. We proceed with the smaller CAS­(10,9) to target the effect of one π* orbital mixing with the d-orbitals. All prior active space formulations enabled the treatment of π-back-donation by incorporating unoccupied π* orbitals localized on the bipyridine ligand. Active spaces that belong to this category are labeled as metal–ligand charge transfer (MLCT) in this work.

Active spaces that do not contain any π* orbitals are called d–d active spaces as they allow only d to d transitions and exclude d to π* transitions or MLCT. With a state-specific orbital optimization procedure, our active spaces do not contain orbitals with π* character on the bpy ligand. By comparing simulated XAS spectra with these two sets of optimized orbitals (d–d active space vs MLCT active space) with the experimental spectra, we can determine the transitions that likely contribute to the shoulder observed in the Ni^II^ XAS. This assessment of active space composition for assigning individual spectral features to specific electronic excitations is analogous to approaches employed in prior theoretical investigations.^26^ By varying active space composition, we reveal the intricacies of core-level excitations in these MC transition-metal systems.

## Computational Methods

All calculations are performed in the OpenMolcas software package version 24.06.^27^ Optimized geometries for **1B**, **1D**, **5D**, and **5B** were obtained from Cagan and co-workers.^16^

We optimize the orbitals of the ground state for each compound using CASSCF^28,29^ with 10 electrons and 9 orbitals. We use the ANO-RCC-VTZP basis set^30,31^ on all atoms and add relativistic effects through the inclusion of terms from the Douglas–Kroll– Hamiltonian and atomic mean-field integrals (AMFI).^32,33^ Default optimization parameters were utilized. In contrast to previous studies, the d–d active spaces are calculated with a state-specific wave function. In our initial guess, we localize the orbitals using the Pipek–Mezey procedure.^34^ We rotate the 3d-orbitals and σ/σ* orbitals into our active space using the ALTER keyword and do not bias the starting unoccupied orbitals toward π* character. The final optimized orbitals for the ground state for each system contained only second shell d-orbitals.

For the MLCT active spaces, we use a state-average wave function, and we include as many states as needed to reliably rotate one π* orbital into the active space. The π* orbital replaces an unoccupied second shell d*_yz_*_/_ *_xz_* ′ orbital, and all other orbitals from the state-specific active space are retained. Orbitals for both active spaces and all compounds are shown in the Supporting Information (SI) in Figures S1–S8. Wave function decomposition for all CASSCF calculations is reported in the SI in Sections S1 and S2.

The core-excited states were generated with HEXS^20^ and RASCI. This approach restricts the full occupation of the RAS1 space and targets core-excitations without the need to use hundreds of excited states and has been used previously to study cobalt bpy complexes.^35^ In our calculations, RAS1 contained three 2p orbitals centered on the nickel, RAS2 contained the d–d or MLCT active space, and RAS3 was left empty. For each RASCI calculation, the orbitals are obtained from either the d–d active space or the MLCT active space. There was 1 hole allowed in RAS1.

Three RASCI wave functions were mixed with the RASSI module : a state-specific ground state, a 15 state average with singlets, and a 15 state average with triplets. Corrections to the energies were included through MC-PDFT^36^ which has been shown to perform relatively well for XAS.^21^ The translated functional was tPBE.^37^ All spectra are convoluted using a Lorentzian broadening function of 0.7 eV (full width at half-maximum (fwhm)). All energies are shifted by −3.7 eV. Previous studies have used a similar or larger shifts^38,39^ and have attributed these errors to a lack of orbital relaxation. We believe this to be the case for our purposes as well. Peak heights were normalized to the maximum peak intensity for each compound.

## Results and Discussion

Because the d–d and MLCT active spaces differ in both orbital composition and CASSCF optimization procedure (state-specific vs state-averaged), we note qualitative differences between the predicted spectral features and discuss overall trends in character.

Experimental^11^ and simulated L_3_-edge spectra for all compounds are shown in Figure 1 in rows (b) and (c). In the d–d active space, **1D** and **1B** show a pre-edge feature red-shifted by approximately 0.7 eV, matching the experimental shoulder (0.9 eV splitting). Compounds **5D** and **5B** lack a shoulder, with spin–orbit states separated by only 0.1 eV, merging the peaks. For all compounds, MLCT active spaces likewise yield a 0.1 eV splitting with no resolved shoulder. The labels peak A and peak B are retained for all compounds as two spin–orbit states contribute to each L_3_-edge despite the misaligned splitting.

What differentiates **1D** and **1B** in the d–d active space from all other calculations is that the spin–orbit states responsible for peak A and peak B have dominant spin-free states with different spin multiplicities. In both compounds, the lowest energy spin-free triplet excited state represents 68% of the spin–orbit state responsible for peak A and the lowest energy spin-free singlet excited state represents 68% of the spin–orbit state responsible for peak B. For all other species, peak A and peak B are dominated by spin-free states with the same spin multiplicity. In Section S3 of the SI we report weights and identities of all spin-free states that contribute to the spin–orbit states responsible for both peak A and peak B in each compound.

The experimental spectra exhibit a systematic decrease in peak A intensity upon ligand substitution, following the trend **5B** < **5D** < **1B** < **1D**. To assess the ability of different active spaces to reproduce this behavior, peak A/peak B oscillator-strength ratios were calculated for both d–d and MLCT active spaces and compared with the experimental intensity ratios shown in Table 1. Relative heights between peak A and peak B for all compounds and active spaces are shown in Figure 1. The MLCT active space more accurately captures the experimental trend (**5D** < **5B** < **1D** < **1B**) than the d–d active space (**1B** < **1D** < **5B** < **5D**), demonstrating the importance of metal–ligand charge transfer character in determining relative peak intensities.

**Table 1:** Qualitative Comparison between the Experimental and Simulated Peak Intensities at the L_3_-Edge^a^

species	$\frac{\mathrm{peak}\;A}{\mathrm{peak}\;B}$ d–d	$\frac{\mathrm{peak}\;A}{\mathrm{peak}\;B}$ MLCT	$\frac{\mathrm{peak}\;A}{\mathrm{peak}\;B}$ exp
1D	0.27	1.1	1.1
1B	0.26	1.2	0.71
5D	0.93	0.84	0.54
5B	0.93	0.98	0.42

a As a proxy for peak-height ratios in the simulations, columns 2 and 3 list oscillator-strength ratios obtained from CASSCF/RASCI­(HEXS)/RASSI using d–d and MLCT active spaces, respectively. A ratio greater than 1 indicates that the spin-orbit state associated with peak A has a higher oscillator strength than the state associated with peak B. Column 4 reports the corresponding peak-height ratio from the experimental spectra.

The two active space treatments yield very different ground-state wave functions. While all of our ground states are spin singlets, the MLCT active space ground states have significant contributions from states that include occupied π* and σ* orbitals which are not present in ground states of the d–d spaces. For the d–d active spaces the ground states have small MC character. We emphasize that the significant spectral differences observed between d–d and MLCT active spaces arise primarily from the inclusion of the π* orbital rather than the removal of the second shell d-orbital, as previous studies on iron complexes have demonstrated that excluding second shell d-orbitals does not substantially alter the L-edge spectral character.^26,40^

For all compounds and active spaces, the spin–orbit states responsible for peak A and peak B are dominated by a spin-free state with an excitation from a 2p orbital into the d_*x* ^2^–*y* ^2^_/σ* orbital. In the d–d active spaces, this configuration accounts for at least 60% of the state, while in the MLCT active spaces it remains the leading contribution at 30% or higher. This assignment is consistent with the expected core–valence excitation of the Ni center. The persistence of the d_*x* ^2^–*y* ^2^_/σ* character across different active spaces highlights its reliability as a spectroscopic marker for Ni^II^ complexes. Additional shakeup contributions (variable occupation of the d-orbitals) with a doubly occupied σ* orbital are present, but none involve direct promotion into the π* orbital, except in the case of compound **1B** with a MLCT active space. In this state, excitation into a π* orbital contributes 19% to the most dominant spin-free state.

Although **1D** contains electron-withdrawing substituents (methyl acetate on bipyridine and methyl on the aryl group) that should promote metal-to-ligand charge transfer, the d–d active space calculation for **1D** successfully reproduces the experimental peak energy splitting but underestimates the peak A/peak B intensity ratio (Table 1). In contrast, the MLCT active space calculation captures the experimental intensity ratio much better, yet neither peak A nor peak B involves direct core-to-π* excitations in the dominant spin-free states. This suggests that the spectroscopic signatures previously attributed to charge transfer arise from indirect effects, such as how π* orbitals in the active space modify d–d transition intensities, rather than from direct 2p → π* excitations. We also note that the L_3_-edge for these compounds are similar to the results found by Flach et al. for Ni^+^ cations in the 3d^8^4s^1 4^F_9/2_ electron configuration and [NiF]^+^ where only 3d localized orbitals are populated in the final states.

Satellite features visible in the experimental spectra are also replicated in both d–d and MLCT simulations. In experiment and simulation the satellite features are blue-shifted from the L_3_ peak by about 2 eV.^11^ The spin-free states that contribute to these satellite features are highly MC in character and are shakeup excitations where core transitions couple to valence d-electron rearrangements. The satellite features are more pronounced in the MLCT states, and **1B** is the only compound that includes a spin-free state with an excitation into the π* orbital. This occurs in feature at 858.6 eV and involves a spin-free state with a 12% contribution to the spin–orbit state. Detailed weights and identities of all spin-free states that contribute to these satellite features are reported in SI Section S3.

## Conclusions

In this work, we used multireference methods to interrogate the origins of the unusual shoulder feature observed in the Ni L_3_-edge spectra of photoredox-active Ni­(bpy)­ArCl complexes by comparing spectra calculated using d–d and MLCT active spaces. The agreement between CASSCF/RASCI­(HEXS)/RASSI simulations and the experiment provides compelling theoretical evidence that configuration mixing in the ground state underlies the anomalous spectral behavior.

We find that only d–d active spaces are able to reproduce the experimental energy splitting between peaks in the L_3_-edge in complexes **1B** and **1D**. This splitting is a result of spin–orbit states dominated by spin-free states with different spin multiplicities, underscoring the role of spin-state mixing in shaping the Ni L_3_-edge. MLCT active spaces, while less successful at resolving the peak splitting, perform better at capturing experimental peak intensity trends. This suggests that the peak positions are governed primarily by d–d transitions, whereas the relative peak heights are sensitive to metal–ligand mixing, supporting previous assignments.^11^

Our analysis also indicates that the experimental shoulder is unlikely a result of a direct 2p → π* excitation. It instead arises from d–d transitions that are perturbed by nearby π* orbitals, highlighting an indirect but significant role for ligand-centered orbitals in modulating spectral shape. The appearance of shakeup features in all active spaces further emphasizes the need for a MC description of both the ground and excited states. We also find that the L_3_ splitting arises from the SOC mixing of states with different multiplicities, while the intensity trend across the series is likely governed by MLCT-driven configuration mixing. This computational investment yields a more nuanced assignment than the semiempirical method, initially used to supplement the experimentally determined spectra.^11^

While the present study establishes components required to reproduce the experimental shoulder, future work with expanded active spaces, using density matrix renormalization^41^ (DMRG) methods, will capture effects that arise from the d–d and MLCT character in one active space. We anticipate that including more orbitals (full set of d-orbitals and balancing π/π* orbitals) in our expanded active space will better reproduce the spectra by properly addressing the static electron correlation. The current approach nonetheless captures the primary characteristics of the observed transitions across both active spaces. The presented results refine the interpretation of L-edge spectral signatures in Ni^II^ photoredox catalysts and establish a foundation for future studies of their excited-state reactivity.

## Supplementary Material



## Notes

Orbital occupation numbers, optimized orbitals, excited-state energies, and dominant contributions to the CI vectors for all wave functions and each compound; decomposition of spin–orbit states that contribute to simulated X-ray absorption spectra (PDF)
