# Supplementary material for: Multireference Modeling Reveals the Origins of L‑Edge X‑ray Absorption Features in Photoredox-Active Nickel Complexes
Source: J Phys Chem C Nanomater Interfaces. 2026 Feb 11;130(8):3034–9. doi: 10.1021/acs.jpcc.6c00223 (PMC12951559; doi:10.1021/acs.jpcc.6c00223)
Supplement: Supplementary file 1 [file jp6c00223_si_001.pdf]

Supporting Information: Multi-reference Modeling Reveals the Origins of L-edge X-ray Absorption Features in Photoredox-Active Nickel Complexes

Olivia Ho[1], Shawna Lin[1], and Thais R. Scott*[1]

[1]Department of Chemistry, Bowdoin College, Brunswick, ME 04011, USA

*Email: t.scott@bowdoin.edu

Contents

S1. CAS(10,9) d-d Wave Function Analysis **S2**

S2. CAS(10,9) MLCT Wave Function Analysis **S5**
Complex 1D S6
Complex 1B S7
Complex 5D S8
Complex 5B S9

S3. X-Ray Absorption Spectra Analysis **S11**
Complex 1D d-d S13
L_3-Edge S13
Satellite Peaks S14
Complex 1B d-d S15
L_3-Edge S15
Satellite Peaks S16
Complex 5D d-d S17
L_3-Edge S17
Satellite Peak S18
Complex 5B d-d S19
L_3-Edge S19
Satellite Peaks S20
Complex 1D MLCT S22
L_3-Edge S22
Satellite Peaks S23
Complex 1B MLCT S25
L_3-Edge S25
Satellite Peaks S26
Complex 5D MLCT S28
L_3-Edge S28
Satellite Peak S29
Complex 5B MLCT S31
L_3-Edge S31
Satellite Peak S32

S1. CAS(10,9) d-d Wave Function Analysis

Final occupation numbers for each species from these state specific CASSCF calculations are reported in table S1. For each species, orbital d_{xy} was nearly doubly occupied. The occupation numbers for the $d_{xz/yz}$ were nearly identical. The d_{z^2} orbitals had occupation numbers lower than the $d_{xz/yz}$ orbitals but higher than the $d_{x^2-y^2}$. The $d_{x^2-y^2}/\sigma$ has the lowest occupation numbers relative to the other doubly occupied natural orbitals. Complex **1D** has the smallest occupation number of 1.947, **1B** follows with 1.950, then **5B** with 1.963 and finally **5D** with 1.967.

The optimized orbitals for each compound are shown in figures S1,S2,S3, and S4. In each case, there are three orbitals that delocalize. The d_{xy} orbital is delocalized but this orbitals does not have significant deviations from an occupation number of 2. The σ and σ^* orbitals both delocalize to cover the bonds between the chlorine and the aryl group. All other orbitals are localized on the Nickel. We label the orbitals $d_{xz/yz}$ twice to indicate that for some systems these orbitals are not perfectly aligned with the XZ or YZ axes and are a linear combination of the two.

The occupation numbers of the $d_{x^2-y^2}/\sigma^*$ orbitals are the highest relative to the other unoccupied natural orbitals with **1D** having the most at 0.070, **1B** next with 0.069, **5D** with 0.066 and finally **5B** with 0.064. This trend in occupation numbers matches the ordering of the height of the L_3-edge shoulder seen in the experimental XAS. The occupation numbers for the $d'_{xz/yz}$ orbitals are nearly identical across all compounds. The d'_{z^2} has a higher occupation number than the orbitals with $d'_{xz/yz}$ character but lower than the $d_{x^2-y^2}/\sigma^*$.

Table S1: Occupation numbers for each species.

Species	$d_{yz/xz}$	$d_{yz/xz}$	d_{z^2}	d_{xy}	$d_{x^2-y^2}/\sigma$	$d_{x^2-y^2}/\sigma^*$	$d'_{yz/xz}$	$d'_{yz/xz}$	d'_{z^2}
1D	1.976	1.973	1.970	1.999	1.947	0.070	0.022	0.019	0.025
1B	1.974	1.977	1.970	1.999	1.950	0.069	0.019	0.018	0.024
5D	1.975	1.973	1.959	1.999	1.967	0.066	0.020	0.018	0.023
5B	1.974	1.973	1.966	1.999	1.963	0.064	0.018	0.019	0.022

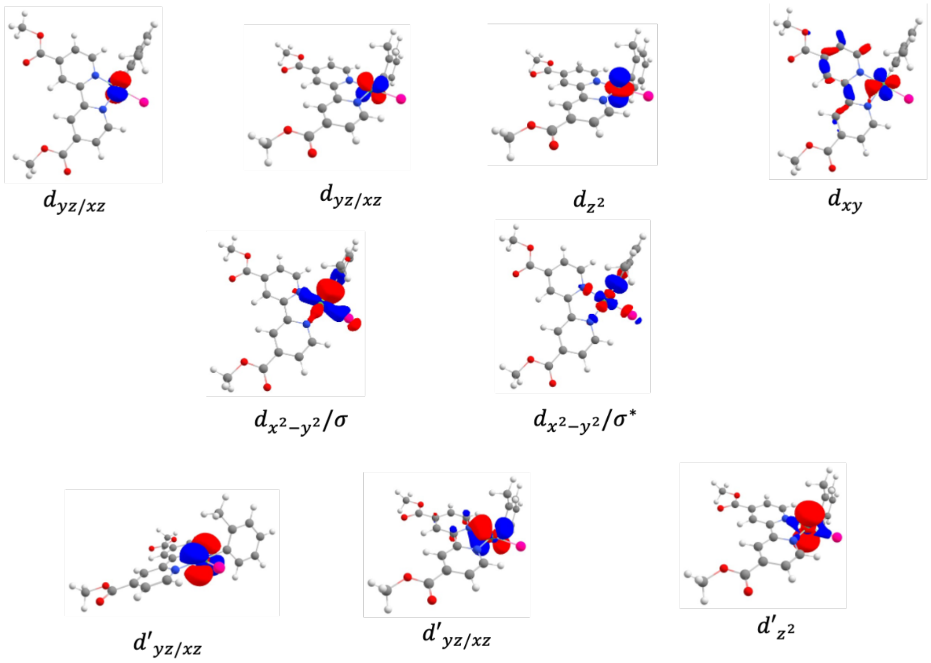

Figure S1: Optimized orbitals included in the CAS(10,9) for compound **1D**.

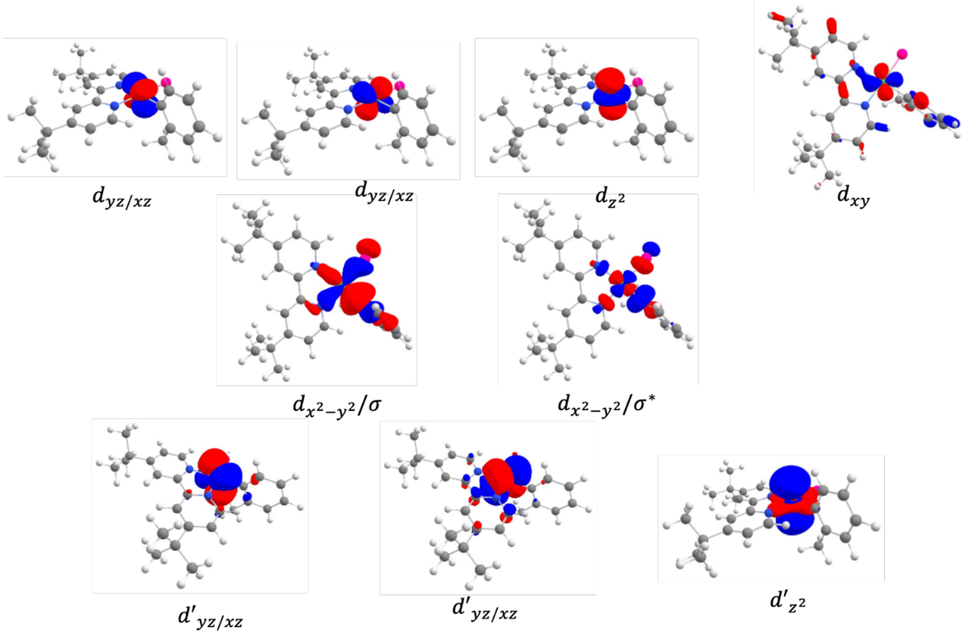

Figure S2: Optimized orbitals included in the CAS(10,9) for compound **1B**.

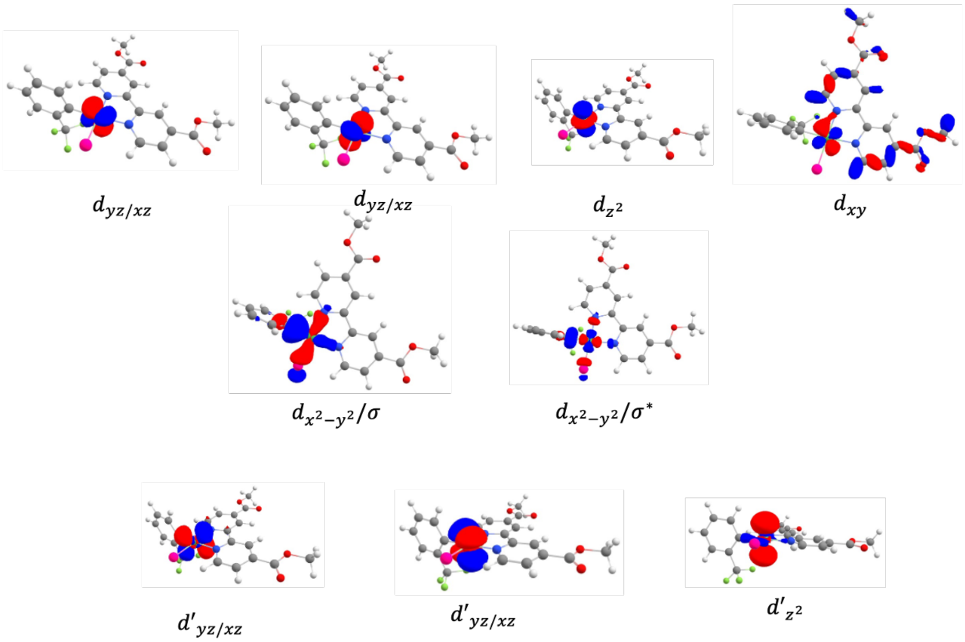

Figure S3: Optimized orbitals included in the CAS(10,9) for compound **5D**.

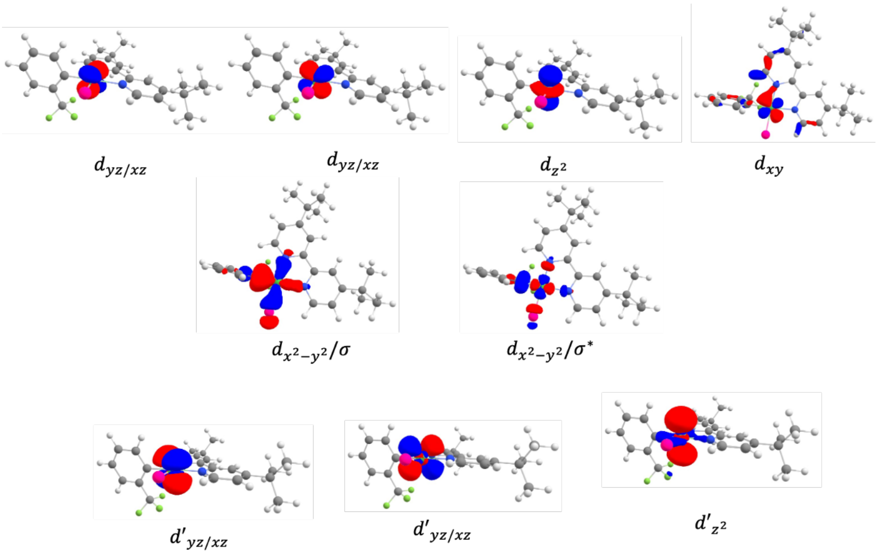

Figure S4: Optimized orbitals included in the CAS(10,9) for compound **5B**.

The CI vectors for the ground state of each complex are reported in tables S2, S3,S4, S5. In each species the dominant configuration is the closed shell singlet with greater than 93% contribution to the wave function.

Table S2: CI vectors with weights greater than 5% for the ground state of **1D**. The order of the orbitals in the CI vector is $d_{yz/xz}$, d_{z^2}, $d_{x^2-y^2}/\sigma$, $d_{yz/xz}$, d_{xy}, $d'_{yz/xz}$, d'_{z^2}, $d_{x^2-y^2}/\sigma^*$, $d'_{yz/xz}$.

CI Vector	Weight	Character
222220000	0.935	Closed shell

Table S3: CI vectors with weights greater than 5% for the ground state of **1B**. The order of the orbitals in the CI vector is $d_{yz/xz}$, $d_{yz/xz}$, d_{z^2}, d_{xy}, $d_{x^2-y^2}/\sigma$, $d_{x^2-y^2}/\sigma^*$, $d'_{yz/xz}$, d'_{z^2}, $d'_{yz/xz}$

CI Vector	Weight	Character
222220000	0.937	Closed shell

Table S4: CI vectors with weights greater than 5% for the ground state of **5D**. The order of the orbitals in the CI vector is $d_{yz/xz}$, d_{z^2}, $d_{yz/xz}$, $d_{x^2-y^2}/\sigma$, d_{xy}, $d'_{yz/xz}$, d'_{z^2}, $d_{x^2-y^2}/\sigma^*$, $d'_{yz/xz}$

CI Vector	Weight	Character
222220000	0.939	Closed shell

Table S5: CI vectors with weights greater than 5% for the ground state of **5B**. The order of the orbitals in the CI vector is $d_{yz/xz}$, d_{z^2}, $d_{yz/xz}$, $d_{x^2-y^2}/\sigma$, $d_{x^2-y^2}/\sigma^*$, d_{xy}, $d'_{yz/xz}$, d'_{z^2}, $d'_{yz/xz}$

CI Vector	Weight	Character
222220000	0.941	Closed shell

S2. CAS(10,9) MLCT Wave Function Analysis

The MLCT active spaces were constructed using restricted state-averaging to include exactly one π^* orbital. This approach avoids known issues with extensive state-averaging (25 triplets and 15 singlets), where energy gap differences between CASSCF and correlated methods can exceed 100 kcal/mol (see Table S8 in the paper by Cagan and coworkers.[1]). While that study employed def2-TZVP/def2-TZVPP basis sets with CPCM solvation, our gas-phase calculations use ano-rcc-vtzp with Douglas–Kroll relativistic corrections. Despite these methodological differences, the fundamental issue of active space instability with extensive state-averaging remains relevant.

Tables S6 and S7 demonstrate this effect for compound **1D**, comparing SA-CASSCF and MC-PDFT energy gaps for 5- and 15-state averages. The maximum deviation (22.36 kcal/mol for the 15-state average) remains below the 35 kcal/mol threshold suggested for active space reliability.[2,3]

The number of states required to rotate in one π^* orbital varied by compound: 5 singlet and 5 triplet states for **1D** and **1B**, 10 singlet states and 10 triplet states for **5D**, and 15 singlet states and 15 triplet states for **5B**. Active space orbitals are shown in Figures S5-S8, with corresponding occupation numbers in Tables S8–S18. The resulting ground-state configurations differ significantly from the d–d active spaces, with dominant configurations listed in Tables S10, S13, S16, and S19 for each compound.

Table S6: Differences in energy gaps between SA-CASSCF and MCPDFT for the 5 state average singlet calculations in 1D.

Root	$\Delta E_{\text{MCPDFT}} - \Delta E_{\text{SA-CASSCF}}$ (kcal/mol)
1	0.00
2	1.27
3	-2.39
4	11.26
5	-12.51

Table S7: Differences in energy gaps between SA-CASSCF and MCPDFT for the 15 state average singlet calculations in 1D.

Root	$\Delta E_{\text{MCPDFT}} - \Delta E_{\text{SA-CASSCF}}$ (kcal/mol)
1	0.00
2	4.64
3	0.78
4	16.47
5	-9.37
6	0.21
7	-2.43
8	-22.36
9	-16.19
10	-14.60
11	2.19
12	12.55
13	-5.13
14	-8.12
15	8.90

Complex 1D

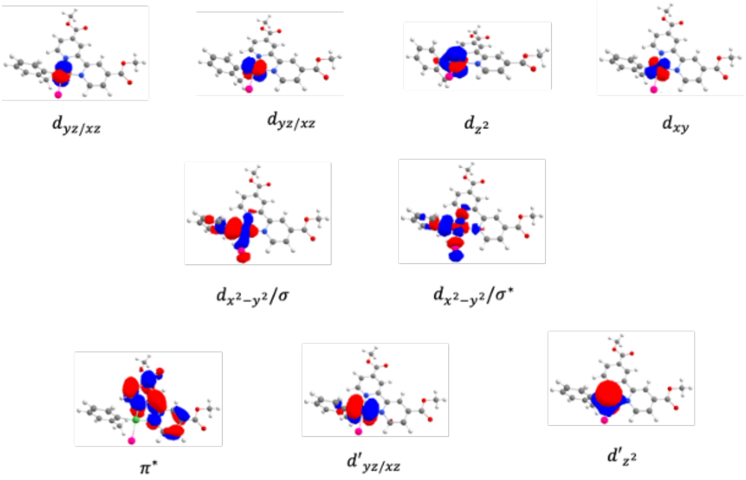

Figure S5: Optimized orbitals included in the CAS(10,9) for simplified compound when 5 singlets/triplets are included in the average.

Table S8: Relative state average CASSCF energies, occupation numbers of each state included in the average for orbital optimization with 5 singlets.

Root	Energy (kcal/mol)	$d_{yz/xz}$	d_{z^2}	$d_{x^2-y^2}/\sigma$	$d_{yz/xz}$	d_{xy}	$d'_{yz/xz}$	d'_{z^2}	$d_{x^2-y^2}/\sigma^*$	π^*
1	0.00	1.978	1.975	1.844	1.626	1.996	0.016	0.019	0.170	0.376
2	23.30	1.984	0.995	1.827	1.998	1.999	0.013	0.007	0.177	1.001
3	29.88	1.311	1.971	1.822	1.979	31.990	0.007	0.017	0.202	0.702
4	34.95	1.939	1.980	1.838	1.955	1.046	0.015	0.018	0.213	0.997
5	52.00	1.972	1.963	1.874	1.842	1.994	0.013	0.021	0.166	0.155

Table S9: Relative state average CASSCF energies, occupation numbers, of each state included in the average for orbital optimization with 5 triplets.

Root	Energy (kcal/mol)	$d_{yz/xz}$	d_{z^2}	$d_{x^2-y^2}/\sigma$	$d_{yz/xz}$	d_{xy}	$d'_{yz/xz}$	d'_{z^2}	$d_{x^2-y^2}/\sigma^*$	π^*
1	12.92	1.011	1.975	1.826	1.977	1.996	0.013	0.015	0.188	0.999
2	17.67	1.995	1.002	1.828	1.983	1.988	0.013	0.011	0.179	1.000
3	28.58	1.977	1.974	1.825	1.018	1.985	0.007	0.013	0.201	1.000
4	44.21	1.900	1.987	1.831	1.927	1.097	0.008	0.014	0.272	0.964
5	50.74	1.064	1.981	1.943	1.898	1.930	0.012	0.016	1.023	0.134

Table S10: CI vectors with weights greater than 5% for the ground state for the state-average calculation for **1D**. The order of the orbitals in the CI vector is $d_{yz/xz}$, d_{z^2} , $d_{x^2-y^2}/\sigma$, $d_{yz/xz}$, d_{xy}, $d'_{yz/xz}$, d'_{z^2},$d_{x^2-y^2}/\sigma^*$, π^*

CI Vector	Weight	Character
222220000	0.837	Closed Shell
22u2200d0	0.094	$d_{x^2-y^2}/\sigma \rightarrow d_{x^2-y^2}/\sigma^*$

Complex 1B

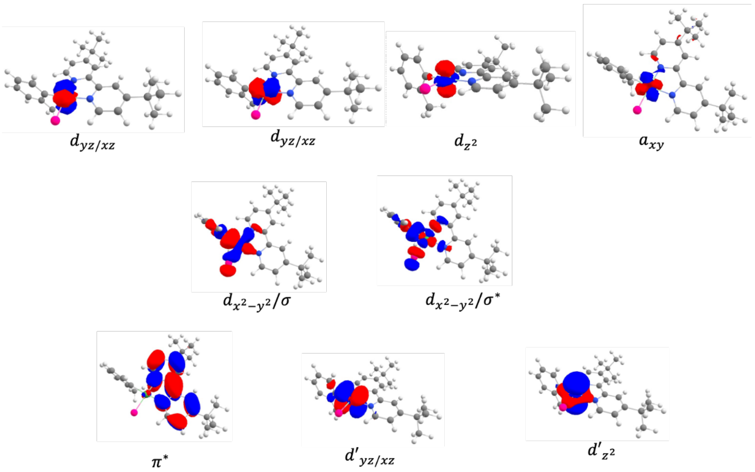

Figure S6: Optimized orbitals included in the CAS(10,9) for simplified compound when 5 singlets/triplets are included in the average.

Table S11: Relative state average CASSCF energies and occupation numbers of each state included in the average for orbital optimization with 5 singlets.

Root	Energy (kcal/mol)	$d_{yz/xz}$	$d_{yz/xz}$	d_{z^2}	d_{xy}	$d_{x^2-y^2}/\sigma$	$d_{x^2-y^2}/\sigma^*$	π^*	d'_{z^2}	$d'_{yz/xz}$
1	0.00	1.905	1.974	1.962	1.997	1.908	0.132	0.085	0.023	0.015
2	34.45	1.402	1.971	1.955	1.995	1.810	0.230	0.602	0.019	0.015
3	37.15	1.998	1.984	0.982	1.995	1.829	0.177	1.014	0.007	0.013
4	50.23	1.988	1.278	1.970	1.994	1.809	0.215	0.721	0.018	0.007
5	64.74	0.999	1.978	1.979	1.999	1.985	1.014	0.005	0.020	0.020

Table S12: Relative state average CASSCF energies and occupation numbers of each state included in the average for orbital optimization with 5 triplets.

Root	Energy (kcal/mol)	$d_{yz/xz}$	$d_{yz/xz}$	d_{z^2}	d_{xy}	$d_{x^2-y^2}/\sigma$	$d_{x^2-y^2}/\sigma^*$	π^*	d'_{z^2}	$d'_{yz/xz}$
1	9.62	0.999	1.986	1.985	2.000	1.995	1.005	0.002	0.015	0.013
2	19.01	1.995	1.988	0.996	1.999	1.993	1.006	0.005	0.006	0.012
3	23.33	1.992	0.996	1.985	2.000	1.992	1.008	0.008	0.014	0.005
4	38.66	1.004	1.985	1.982	1.995	1.838	0.172	0.997	0.015	0.014
5	45.91	1.997	1.985	0.998	1.994	1.844	0.164	1.000	0.007	0.012

Table S13: CI vectors with weights greater than 5% for the ground state of **1B**. The order of the orbitals in the CI vector is d_{yz}, d_{xz},d_{z^2}, d_{xy}, $d_{x^2-y^2}/\sigma$, $d_{x^2-y^2}/\sigma^*$, π, d'_{z^2}, d'_{xz}

CI Vector	Weight	Character
222220000	0.710	Closed Shell
u22220d00	0.130	$d_{yz/xz} \rightarrow \pi^*$

Complex 5D

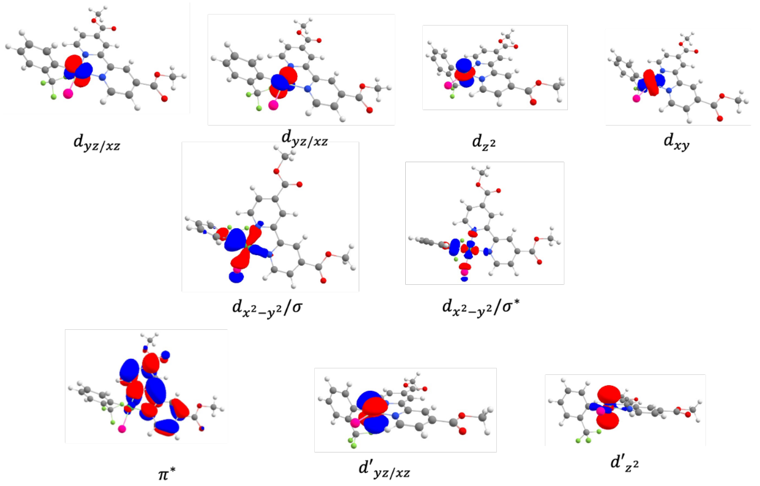

Figure S7: Optimized orbitals included in the CAS(10,9) when 15 singlets are included in the average.

Table S14: Relative state average CASSCF energies and occupation numbers of each state included in the average for orbital optimization with 10 singlets.

Root	Energy (kcal/mol)	$d_{yz/xz}$	d_{xy}	$d_{yz/xz}$	$d_{x^2-y^2}/\sigma$	d_{z^2}	$d'_{yz/xz}$	π^*	$d_{x^2-y^2}/\sigma^*$	d'_{z^2}
1	0.00	1.973	1.997	1.900	1.918	1.966	0.015	0.089	0.122	0.019
2	44.27	1.972	1.994	1.377	1.820	1.956	0.013	0.626	0.226	0.016
3	46.13	1.997	1.983	1.994	1.846	1.133	0.013	0.863	0.164	0.007
4	56.02	1.278	1.904	1.962	1.811	1.976	0.008	0.746	0.300	0.016
5	57.72	1.924	1.205	1.788	1.869	1.980	0.013	0.860	0.343	0.017
6	61.80	1.870	1.116	1.978	1.949	1.971	0.014	0.144	0.942	0.018
7	64.65	1.971	1.893	1.103	1.963	1.983	0.014	0.099	0.957	0.017
8	77.02	1.963	1.992	1.944	1.977	1.052	0.012	0.036	1.017	0.007
9	78.08	1.099	1.895	1.955	1.962	1.984	0.007	0.083	1.000	0.016
10	114.78	1.902	1.097	1.236	1.947	1.880	0.012	0.844	1.068	0.014

Table S15: Relative state average CASSCF energies and occupation numbers of each state included in the average for orbital optimization with 10 triplets.

Root	Energy (kcal/mol)	d_{z^2}	$d_{yz/xz}$	$d_{yz/xz}$	$d_{x^2-y^2}/\sigma$	d_{xy}	$d'_{yz/xz}$	π^*	$d_{x^2-y^2}/\sigma^*$	s d'_{z^2}
1	16.03	1.975	1.990	1.010	1.995	1.984	0.013	0.003	1.016	0.014
2	26.13	0.998	1.997	1.992	1.990	1.986	0.009	0.013	1.009	0.005
3	28.12	1.985	1.987	1.974	1.990	1.009	0.006	0.013	1.022	0.015
4	30.12	1.943	1.047	1.934	1.985	1.984	0.011	0.021	1.060	0.015
5	40.29	1.981	1.993	1.009	1.857	1.978	0.013	0.998	0.155	0.014
6	49.20	0.996	1.999	1.996	1.858	1.985	0.012	1.000	0.148	0.006
7	58.13	1.978	1.987	1.981	1.851	1.011	0.006	0.999	0.173	0.014
8	64.77	1.980	1.061	1.907	1.871	1.926	0.013	0.965	0.262	0.015
9	69.40	1.888	1.094	1.121	1.994	1.895	0.010	0.036	1.948	0.012
10	78.85	1.037	1.036	1.958	1.999	1.953	0.005	0.006	1.995	0.011

Table S16: CI vectors with weights greater than 5% for the ground state of the simplified structure. The order of the orbitals in the CI vector is d_{yz}, d_{xz},d_{z^2}, d_{xy}, $d_{x^2-y^2}/\sigma$, $d_{x^2-y^2}/\sigma^*$, π, d'_{z^2}, d'_{xz}

CI Vector	Weight	Character
222220000	0.729	Closed Shell
22u220d00	0.119	$d_{yz/xz} \rightarrow \pi^*$
222u200d0	0.056	$d_{x^2-y^2}/\sigma \rightarrow d_{x^2-y^2}/\sigma^*$

Complex 5B

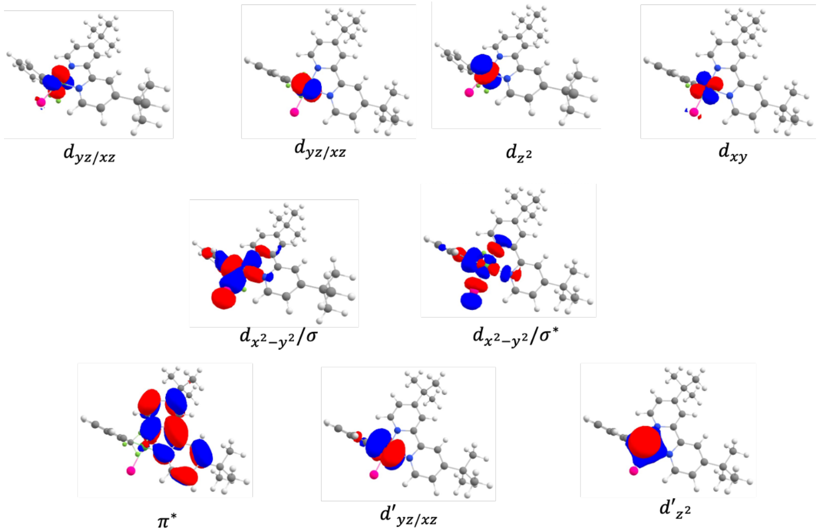

Figure S8: Optimized orbitals included in the CAS(10,9) for simplified compound when 15 singlets/triplets are included in the average.

Table S17: Relative state average CASSCF energies and occupation numbers for each state included in the average for orbital optimization with 15 singlets.

Root	Energy (kcal/mol)	$d_{yz/xz}$	$d_{yz/xz}$	d_{z^2}	$d_{x^2-y^2}/\sigma$	d_{xy}	$d_{x^2-y^2}/\sigma^*$	$d'_{yz/xz}$	π^*	d'_{z^2}
1	0.00	1.972	1.941	1.965	1.949	1.997	0.104	0.014	0.041	0.017
2	52.78	1.973	1.231	1.984	1.902	1.816	0.783	0.013	0.283	0.015
3	54.49	1.985	1.946	1.982	1.973	1.067	0.962	0.013	0.056	0.015
4	55.29	1.969	1.142	1.986	1.931	1.905	0.887	0.013	0.151	0.015
5	58.28	1.983	1.994	1.017	1.858	1.999	0.155	0.012	0.977	0.006
6	65.63	1.166	1.948	1.983	1.914	1.834	0.911	0.007	0.222	0.014
7	67.21	1.762	1.957	1.238	1.911	1.989	0.851	0.012	0.272	0.008
8	68.85	1.301	1.976	1.695	1.873	1.989	0.438	0.008	0.708	0.012
9	75.34	1.755	1.958	1.981	1.865	1.233	0.412	0.011	0.770	0.015
10	109.07	1.946	1.755	1.898	1.999	0.384	1.992	0.011	0.003	0.012
11	115.70	1.927	1.039	1.988	1.998	1.080	1.936	0.011	0.008	0.013
12	115.92	1.741	1.723	1.290	1.999	1.231	1.972	0.008	0.028	0.007
13	124.07	1.875	1.189	1.883	1.956	1.109	1.079	0.011	0.884	0.013
14	127.23	1.052	1.942	1.938	1.999	1.057	1.991	0.005	0.006	0.011
15	131.87	1.981	1.989	0.960	1.992	1.009	1.061	0.011	0.992	0.005

Table S18: Relative state average CASSCF energies and occupation numbers for each state included in the average for orbital optimization with 15 triplets.

Root	Energy (kcal/mol)	$d_{yz/xz}$	$d_{yz/xz}$	d_{z^2}	$d_{x^2-y^2}/\sigma$	d_{xy}	$d_{x^2-y^2}/\sigma^*$	$d'_{yz/xz}$	π^*	d'_{z^2}
1	12.40	1.975	1.011	1.987	1.995	1.988	1.015	0.002	0.014	0.013
2	23.09	1.986	1.995	0.998	1.993	1.998	1.007	0.006	0.005	0.012
3	25.72	1.018	1.970	1.987	1.993	1.977	1.029	0.008	0.013	0.005
4	28.15	1.916	1.922	1.984	1.989	1.070	1.079	0.014	0.015	0.010
5	45.57	1.978	1.008	1.983	1.859	1.993	0.153	0.999	0.014	0.013
6	54.02	1.985	1.996	0.997	1.862	1.998	0.145	1.000	0.006	0.012
7	62.89	1.890	1.105	1.892	1.998	1.099	1.983	0.009	0.012	0.011
8	63.23	1.012	1.980	1.980	1.852	1.985	0.173	0.999	0.014	0.005
9	70.80	1.902	1.926	1.978	1.871	1.073	0.243	0.981	0.015	0.013
10	73.39	1.008	1.987	1.981	1.999	1.010	1.995	0.005	0.005	0.010
11	73.70	1.911	1.919	1.085	1.998	1.079	1.980	0.013	0.010	0.005
12	94.25	1.261	1.077	1.963	1.995	1.734	1.718	0.232	0.012	0.007
13	104.80	1.731	1.218	1.885	1.967	1.264	1.200	0.712	0.013	0.010
14	106.89	1.906	1.924	1.043	1.990	1.084	1.105	0.930	0.007	0.011
15	110.77	1.353	1.663	1.676	1.975	1.336	1.047	0.931	0.011	0.008

Table S19: CI vectors with weights greater than 5% for the ground state of the simplified structure. The order of the orbitals in the CI vector is $d_{yz/xz}$, $d_{xz/yz}$, d_{z^2}, $d_{x^2-y^2}/\sigma$, d_{xy}, $d_{x^2-y^2}/\sigma^*$, d'_{z^2}, π^*, d'_{xz}.

CI Vector	Weight	Character
222220000	0.770	Closed Shell
222u2d000	0.099	$d_{z^2} \rightarrow d_{x^2-y^2}/\sigma^*$
2u22200d0	0.063	$d_{xz/yz} \rightarrow \pi^*$

S3. X-Ray Absorption Spectra Analysis

X-ray absorption spectra were computed using restricted active space configuration interaction (RASCI) calculations followed by state interaction via the restricted active space state interaction (RASSI) module in openmolcas. Three independent RASCI calculations were performed using the orbitals described previously as the reference. The expanded RAS active space was constructed by designating the three core 2p orbitals to the RAS1 subspace, while maintaining the same orbital composition as the CAS(10,9) reference in the RAS2. The inclusion of core orbitals in the RASCI treatment produced negligible changes in the ground-state electronic structure, with orbital occupation numbers differing by at most 0.002 electrons from the corresponding CASSCF calculation.

The three RASCI calculations are as follows: (i) the ground state, (ii) 15 singlet excited states, and (iii) 15 triplet excited states. Core excitations were selectively targeted using the HEXS keyword, which removes the maximum occupation constraint in RAS1. The inclusion of 15 states for each multiplicity ensures complete coverage of both L_3 and L_2 edge transitions. Figure S9 shows our full calculated spectra and figure S10 shows the spin-free spectra. All spectra are convoluted using a Lorentzian broadening function of 0.7 eV (FWHM). All energies are shifted by -3.7 eV. Peak heights were normalized to the max peak intensity for each compound.

Compound **1B** deviates the most from the expected ratio between the L_3 and L_2 edges (Figure S9) and the intensity of the L_2 relative to the L_3-egde is overestimated in all cases. In figures S10 and S11, we also show the spin-free spectra without spin-orbit coupling to highlight its importance in splitting the L_2 and L_3 peaks.

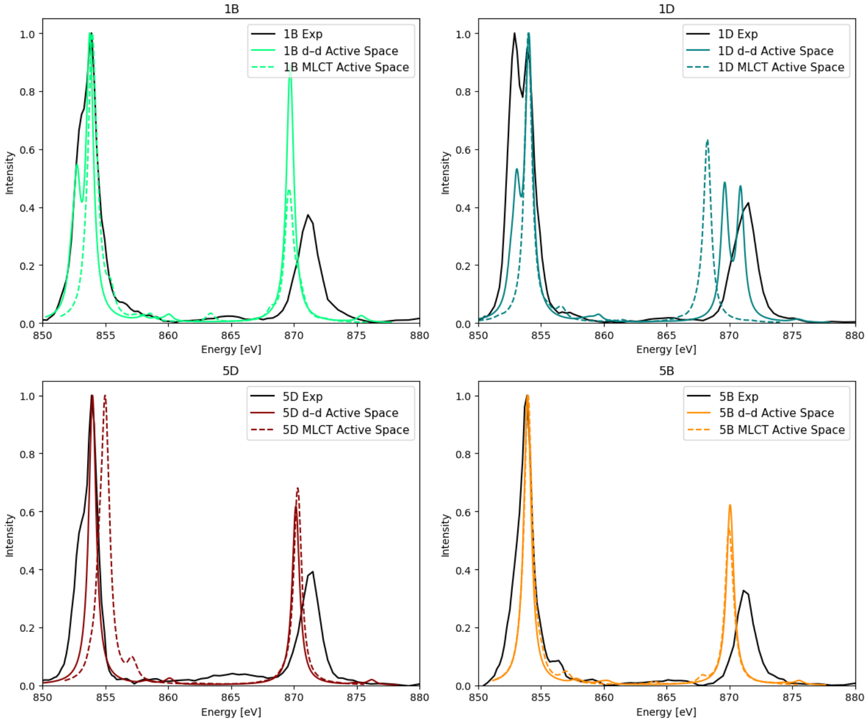

Figure S9: Comparison of experimental[4] and simulated X-ray absorption spectra (this work) at the L_3 and L_2 edge. Each panel shows the experimental spectrum (black, solid line) with two simulated spectra. (i) a calculation using a d–d active space (colored solid line), and (ii) a calculation using a metal-to-ligand charge transfer (MLCT) active space (colored dashed line). All spectra are normalized to unit peak intensity, and simulated spectra are shifted by –3.7 eV to align with experiment and Lorentzian broadened by 0.7 eV.

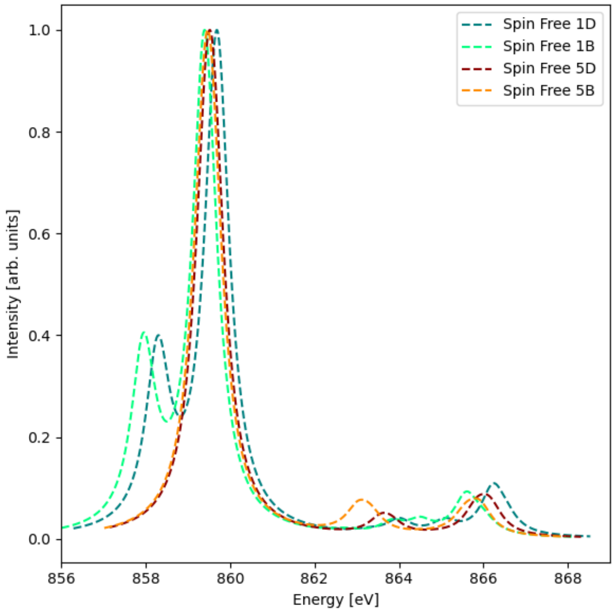

Figure S10: Unshifted spin-free simulated L-edge spectra for all 4 compounds. Orbitals are optimized with a state specific CAS(10,9) wave function.

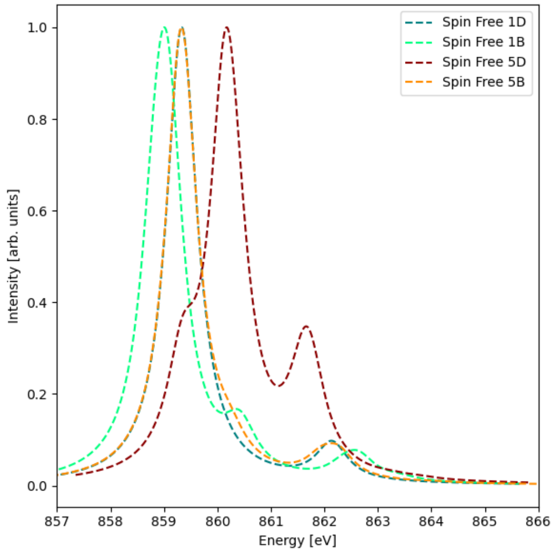

Figure S11: Unshifted spin-free simulated L-edge spectra for all 4 compounds. Orbitals are optimized with a state average CAS(10,9) wave function.

Complex 1D d-d

L_3-Edge

Table S20: The spin free states that contribute at least 5% to peak A in **1D**. The order of the orbitals in the CI vector is p_y, p_z, p_x, $d_{yz/xz}$, d_{z^2}, $d_{x^2-y^2}/\sigma$, $d_{yz/xz}$, d_{xy}, $d'_{yz/xz}$, d'_{z^2}, $d_{x^2-y^2}/\sigma^*$, $d'_{yz/xz}$.

Spin-free State	Spin	Weight	CI Vectors (Weights)
17	1.0	0.678	22u2222200u0 (0.855)
19	1.0	0.259	2u22222200u0 (0.836)
4	0.0	0.059	u222222200d0 (0.728)<br>u2222d220020 (0.085)<br>u222d2220020 (0.053)

Table S21: The spin free states that contribute at least 5% to peak B in **1D**. The order of the orbitals in the CI vector is p_y, p_z, p_x, $d_{yz/xz}$, d_{z^2}, $d_{x^2-y^2}/\sigma$, $d_{yz/xz}$, d_{xy}, $d'_{yz/xz}$, d'_{z^2}, $d_{x^2-y^2}/\sigma^*$, $d'_{yz/xz}$.

Spin-free State	Spin	Weight	CI Vectors (Weights)
2	0.0	0.680	22u2222200d0 (0.747)<br>22u22d220020 (0.109)
19	1.0	0.181	2u22222200u0 (0.836)
18	1.0	0.127	u222222200u0 (0.849)

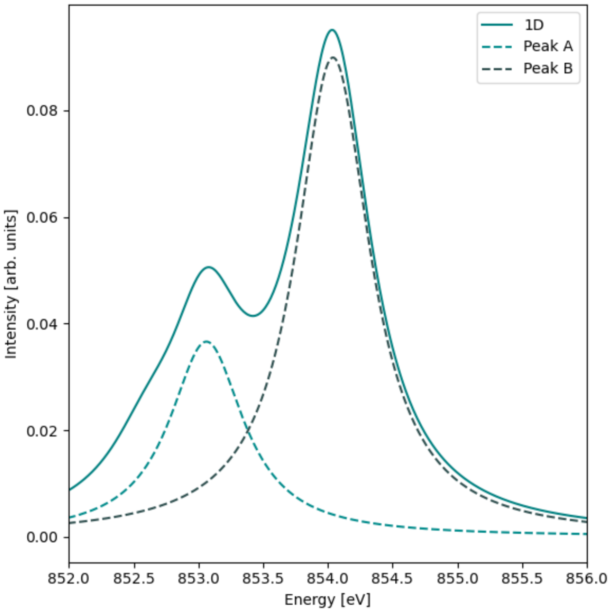

Figure S12: Contributions to the L_3 edge for 1D.

Satellite Peaks

Table S22: The spin free states that contribute at least 10% to the feature at 858.5 eV in **1D**. The order of the orbitals in the CI vector is p_y, p_z, p_x, $d_{yz/xz}$, d_{z^2}, $d_{x^2-y^2}/\sigma$, $d_{yz/xz}$, d_{xy}, $d'_{yz/xz}$, d'_{z^2}, $d_{x^2-y^2}/\sigma^*$, $d'_{yz/xz}$.

Spin-free State	Spin	Weight	CI Vectors (Weights)
20	1	0.264	22uu22220020 (0.756)
8	0	0.201	2u22222d0020 (0.178)
22	1	0.186	u22d22220020 (0.550)
26	1	0.178	u22u22220020 (0.776)

Table S23: The spin free states that contribute at least 10% to the feature 858.9 eV in **1D**. The order of the orbitals in the CI vector is p_y, p_z, p_x, $d_{yz/xz}$, d_{z^2}, $d_{x^2-y^2}/\sigma$, $d_{yz/xz}$, d_{xy}, $d'_{yz/xz}$, d'_{z^2}, $d_{x^2-y^2}/\sigma^*$, $d'_{yz/xz}$.

Spin-free State	Spin	Weight	CI Vectors (Weights)
25	1	0.386	22u22u220020 (0.723)<br>2u2u22220020 (0.111)
26	1	0.101	u22u22220020 (0.776)

Table S24: The spin free states that contribute at least 10% to the feature at 859.6 eV in **1D**. The order of the orbitals in the CI vector is p_y, p_z, p_x, $d_{yz/xz}$, d_{z^2}, $d_{x^2-y^2}/\sigma$, $d_{yz/xz}$, d_{xy}, $d'_{yz/xz}$, d'_{z^2}, $d_{x^2-y^2}/\sigma^*$, $d'_{yz/xz}$.

Spin-free State	Spin	Weight	CI Vectors (Weights)
16	0	0.257	u222222200d0 (0.106)<br>2u2222d20020 (0.183)<br>u2222d220020 (0.546)
30	1	0.189	u22222u20020 (0.307)<br>2u22u2220020 (0.492)
27	1	0.183	22u22u220020 (0.138)<br>2u2u22220020 (0.596)
23	1	0.109	2u2222u20020 (0.405)<br>u222u2220020 (0.364)

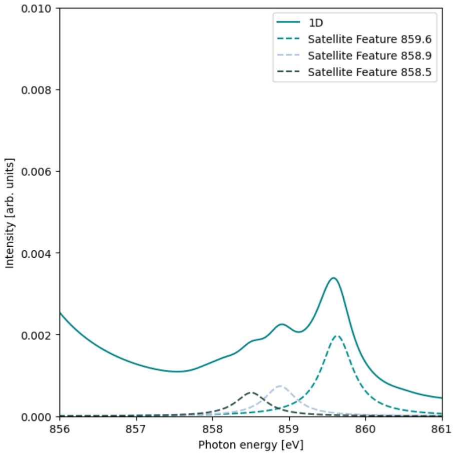

Figure S13: Contributions to the satellite peaks for **1D**. Lorentzian broadened with gamma 0.7.

Complex 1B d-d

L_3-Edge

Table S25: The spin free states that contribute at least 5% to peak A in **1B**. The order of the orbitals in the CI vector is p_x, p_z, p_y, $d_{yz/xz}$, $d_{yz/xz}$, d_{z^2}, d_{xy}, $d_{x^2-y^2}/\sigma$, $d_{x^2-y^2}/\sigma^*$, $d'_{yz/xz}$, d'_{z^2}, $d'_{yz/xz}$

Spin-free State	Spin	Weight	CI Vectors (Weights)
17	1.0	0.680	u2222222u000 (0.861)
19	1.0	0.255	2u222222u000 (0.838)
4	0.0	0.062	22u22222d000 (0.732)
			22u2222d2000 (0.085)
			22u22d222000 (0.058)

Table S26: The spin free states that contribute at least 5% to peak B in **1B**. The order of the orbitals in the CI vector is p_x, p_z, p_y, $d_{yz/xz}$, $d_{yz/xz}$, d_{z^2}, d_{xy}, $d_{x^2-y^2}/\sigma$, $d_{x^2-y^2}/\sigma^*$, $d'_{yz/xz}$, d'_{z^2}, $d'_{yz/xz}$

Spin-free State	Spin	Weight	CI Vectors (Weights)
2	0.0	0.684	u2222222d000 (0.748)
			u222222d2000 (0.108)
			22u22d222000 (0.058)
19	1.0	0.172	2u222222u000 (0.838)
18	1.0	0.132	22u22222u000 (0.856)

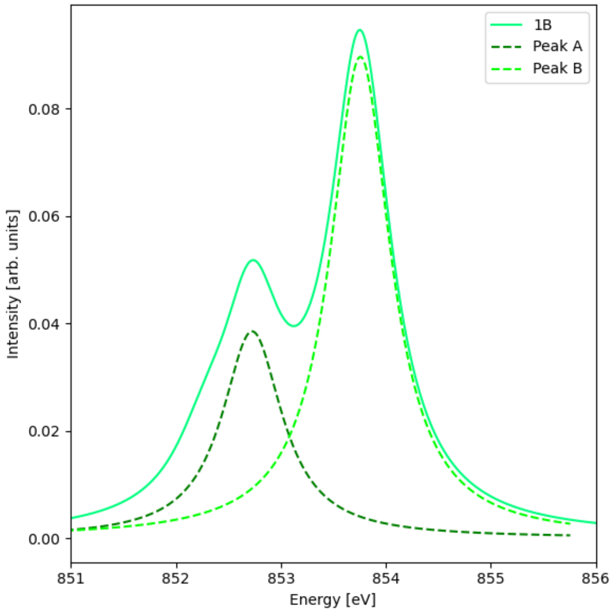

Figure S14: Contributions to the L_3 edge for **1B**. Lorentzian broadened with gamma 0.7.

Satellite Peaks

Table S27: The spin free states that contribute at least 10% to feature at 858.0 eV in **1B**. The order of the orbitals in the CI vector is p_x, p_z, p_y, $d_{yz/xz}$, $d_{yz/xz}$, d_{z^2}, d_{xy}, $d_{x^2-y^2}/\sigma$, $d_{x^2-y^2}/\sigma^*$, $d'_{yz/xz}$, d'_{z^2}, $d'_{yz/xz}$

Spin-free State	Spin	Weight	CI Vectors (Weights)
22	1	0.3244	u2222u222000 (0.838)
20	1	0.172	u222u2222000 (0.737)
8	0	0.144	22u2222d2000 (0.216)<br>22u22d222000 (0.434)<br>2u2d22222000 (0.152)

Table S28: The spin free states that contribute at least 10% to feature at 859.0 eV in **1B**. The order of the orbitals in the CI vector is p_x, p_z, p_y, $d_{yz/xz}$, $d_{yz/xz}$, d_{z^2}, d_{xy}, $d_{x^2-y^2}/\sigma$, $d_{x^2-y^2}/\sigma^*$, $d'_{yz/xz}$, d'_{z^2}, $d'_{yz/xz}$

Spin-free State	Spin	Weight	CI Vectors (Weights)
10	0	0.528	u2222222d000 (0.112)<br>u222222d2000 (0.664)
31	1	0.296	2u22222u2000 (0.880)

Table S29: The spin free states that contribute at least 10% to feature at 860.0 eV in **1B**. The order of the orbitals in the CI vector is p_x, p_z, p_y, $d_{yz/xz}$, $d_{yz/xz}$, d_{z^2}, d_{xy}, $d_{x^2-y^2}/\sigma$, $d_{x^2-y^2}/\sigma^*$, $d'_{yz/xz}$, d'_{z^2}, $d'_{yz/xz}$

Spin-free State	Spin	Weight	CI Vectors (Weights)
15	0	0.322	22u2222d2000 (0.471)<br>2u2d22222000 (0.208)
31	1	0.298	2u22222u2000 (0.880)
13	0	0.165	22u22d222000 (0.332)<br>2u2d22222000 (0.454)

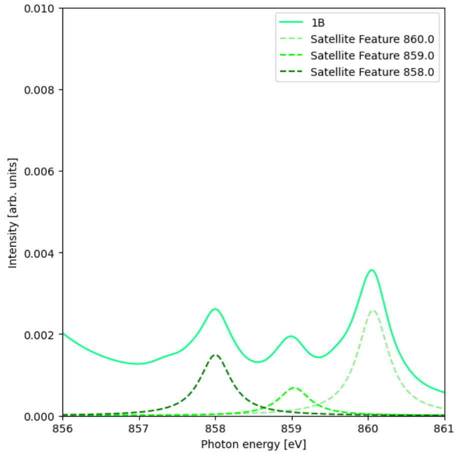

Figure S15: Contributions to the satellite peaks for **1B**. Lorentzian broadened with gamma 0.7.

Complex 5D d-d

L_3-Edge

Table S30: The spin free states that contribute at least 5% to peak A in **5D**. The order of the orbitals in the CI vector is p_x, p_y, p_z, $d_{yz/xz}$, d_{xy}, $d_{yz/xz}$, $d_{x^2-y^2}/\sigma$, d_{z^2}, $d'_{yz/xz}$, $d'_{yz/xz}$, $d_{x^2-y^2}/\sigma^*$, d'_{z^2}.

Spin-free State	Spin	Weight	CI Vectors (Weights)
3	0.0	0.633	2u22222200d0 (0.722)<br>2u22222d0020 (0.080)<br>2u2222d20020 (0.063)
18	1.0	0.247	u222222200u0 (0.835)
19	1.0	0.096	22u2222200u0 (0.841)

Table S31: The spin free states that contribute at least 5% to peak B in **5D**. The order of the orbitals in the CI vector is p_x, p_y, p_z, $d_{yz/xz}$, d_{z^2}, $d_{yz/xz}$, $d_{x^2-y^2}/\sigma$, d_{xy}, $d'_{yz/xz}$, d'_{z^2}, $d_{x^2-y^2}/\sigma^*$, $d'_{yz/xz}$

Spin-free State	Spin	Weight	CI Vectors (Weights)
4	0.0	0.634	u222222200d0 (0.722)<br>u222222d0020 (0.071)<br>u22222d20020 (0.074)
17	1.0	0.205	2u22222200u0 (0.848)
19	1.0	0.133	22u2222200u0 (0.841)

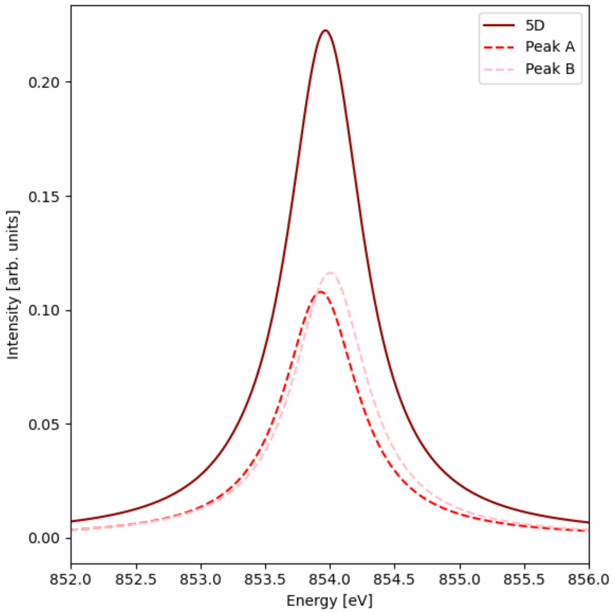

Figure S16: Contributions to the L_3 edge for **5D**. Lorentzian broadened with gamma 0.7.

Satellite Peak

Table S32: The spin free states that contribute at least 10% to feature at 858.2 eV in **5D**. The order of the orbitals in the CI vector is p_x, p_y, p_z, $d_{yz/xz}$, d_{z^2}, $d_{yz/xz}$, $d_{x^2-y^2}/\sigma$, $d_{x^2-y^2}/\sigma^*$, d_{xy}, $d'_{yz/xz}$, d'_{z^2}, $d'_{yz/xz}$

Spin-free State	Spin	Weight	CI Vectors (Weights)
6	0.0	0.524	u222222d0020 (0.491)
			u22222d20020 (0.126)
			22ud22220020 (0.153)
28	1.0	0.220	22u2222u0020 (0.376)
			2u222u220020 (0.214)
			u22u22220020 (0.195)
24	1.0	0.1391	2u222u220020 (0.328)
			u22u22220020 (0.454)

Table S33: The spin free states that contribute at least 10% to feature at 859.2 eV in **5D**. The order of the orbitals in the CI vector is p_x, p_y, p_z, $d_{yz/xz}$, d_{z^2}, $d_{yz/xz}$, $d_{x^2-y^2}/\sigma$, $d_{x^2-y^2}/\sigma^*$, d_{xy}, $d'_{yz/xz}$, d'_{z^2}, $d'_{yz/xz}$

Spin-free State	Spin	Weight	CI Vectors (Weights)
10	0.0	0.574	2u222d220020 (0.376)
			u22d22220020 (0.309)
21	1.0	0.127	2u22222u0020 (0.399)
			22u22u220020 (0.430)
22	1.0	0.119	u222222u0020 (0.352)
			22uu22220020 (0.475)

Table S34: The spin free states that contribute at least 10% to feature at 860.2 eV in **5D**. The order of the orbitals in the CI vector is p_x, p_y, p_z, $d_{yz/xz}$, d_{z^2}, $d_{yz/xz}$, $d_{x^2-y^2}/\sigma$, $d_{x^2-y^2}/\sigma^*$, d_{xy}, $d'_{yz/xz}$, d'_{z^2}, $d'_{yz/xz}$

Spin-free State	Spin	Weight	CI Vectors (Weights)
16	0.0	0.596	u222222200d0 (0.102)
			u22222d20020 (0.715)
31	1.0	0.254	22u222u20020 (0.895)

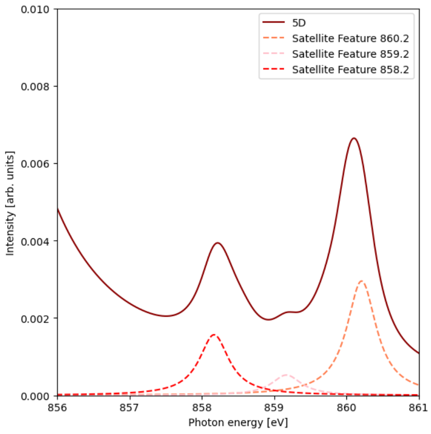

Figure S17: Contributions to the satellite peaks for **5D**. Lorentzian broadened with gamma 0.7.

Complex 5B d-d

L_3-Edge

Table S35: The spin free states that contribute at least 5% to peak A in **5B**. The order of the orbitals in the CI vector is p_y, p_z, p_x, $d_{yz/xz}$, $d_{yz/xz}$, d_{z^2}, $d_{x^2-y^2}/\sigma$, d_{xy}, $d_{x^2-y^2}/\sigma^*$, $d'_{yz/xz}$, d'_{z^2}, $d'_{yz/xz}$

Spin-free State	Spin	Weight	CI Vectors (Weights)
3	0.0	0.627	2u222222d000 (0.706)<br>2u2222d22000 (0.058)<br>2u222d222000 (0.089)
18	1.0	0.248	22u22222u000 (0.825)
19	1.0	0.092	u2222222u000 (0.824)

Table S36: The spin free states that contribute at least 5% to peak B in **5B**. The order of the orbitals in the CI vector is p_y, p_z, p_x, $d_{yz/xz}$, $d_{yz/xz}$, d_{z^2}, $d_{x^2-y^2}/\sigma$, d_{xy}, $d_{x^2-y^2}/\sigma^*$, $d'_{yz/xz}$, d'_{z^2}, $d'_{yz/xz}$.

Spin-free State	Spin	Weight	CI Vectors (Weights)
4	0.0	0.626	22u22222d000 (0.705)<br>22u222d22000 (0.051)<br>22u22d222000 (0.098)
17	1.0	0.211	u2222222d000 (0.798)
19	1.0	0.124	u2222222u000 (0.824)

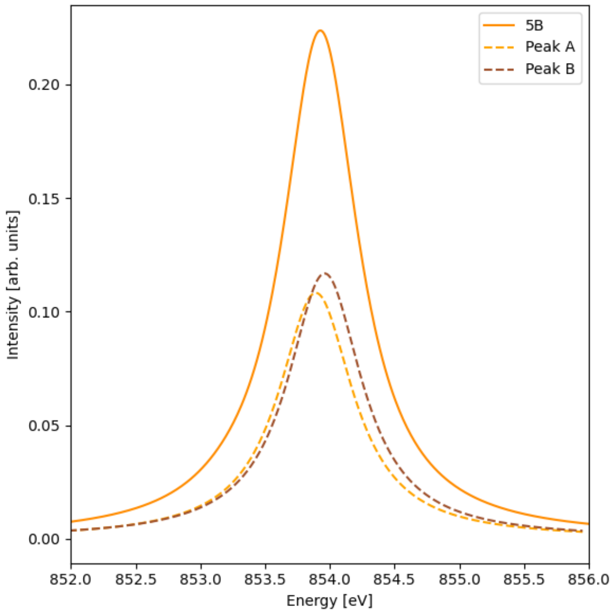

Figure S18: Contributions to the L_3 edge for **5B**. Lorentzian broadened with gamma 0.7.

Satellite Peaks

Table S37: The spin free states that contribute at least 10% to feature at 857.8 eV in **5B**. The order of the orbitals in the CI vector is p_y, p_z, p_x, $d_{yz/xz}$, $d_{yz/xz}$, d_{z^2}, $d_{x^2-y^2}/\sigma$, d_{xy}, $d_{x^2-y^2}/\sigma^*$, $d'_{yz/xz}$, d'_{z^2}, $d'_{yz/xz}$.

Spin-free State	Spin	Weight	CI Vectors (Weights)
24	1.0	0.335	2u22u2222000 (0.343)<br>22uu22222000 (0.467)
6	0.0	0.304	22u22u222000 (0.319)<br>u22u22222000 (0.484)
28	1.0	0.143	u2222u222000 (0.372)<br>2u22u2222000 (0.232)<br>22uu22222000 (0.212)
23	1.0	0.131	22u2u2222000 (0.311)<br>2u2u22222000 (0.511)

Table S38: The spin free states that contribute at least 10% to feature at 858.8 eV in **5B**. The order of the orbitals in the CI vector is p_y, p_z, p_x, $d_{yz/xz}$, $d_{yz/xz}$, d_{z^2}, $d_{x^2-y^2}/\sigma$, d_{xy}, $d_{x^2-y^2}/\sigma^*$, $d'_{yz/xz}$, d'_{z^2}, $d'_{yz/xz}$.

Spin-free State	Spin	Weight	CI Vectors (Weights)
10	0.0	0.570	2u22d2222000 (0.406)<br>22ud22222000 (0.344)
21	1.0	0.126	2u222u222000 (0.353)<br>u222u2222000 (0.441)
27	1.0	0.101	22u22u222000 (0.152)<br>2u222u222000 (0.314)<br>u222u2222000 (0.393)
22	1.0	0.101	22u22u222000 (0.319)<br>u22u22222000 (0.484)

Table S39: The spin free states that contribute at least 10% to feature at 859.7 eV in **5B**. The order of the orbitals in the CI vector is p_y, p_z, p_x, $d_{yz/xz}$, $d_{yz/xz}$, d_{z^2}, $d_{x^2-y^2}/\sigma$, d_{xy}, $d_{x^2-y^2}/\sigma^*$, $d'_{yz/xz}$, d'_{z^2}, $d'_{yz/xz}$.

Spin-free State	Spin	Weight	CI Vectors (Weights)
31	1.0	0.375	u22222u22000 (0.850)
13	0.0	0.227	u2222d222000 (0.765)

Table S40: The spin free states that contribute at least 10% to the feature at 860.4 eV in **5B**. The order of the orbitals in the CI vector is p_y, p_z, p_x, $d_{yz/xz}$, $d_{yz/xz}$, d_{z^2}, $d_{x^2-y^2}/\sigma$, d_{xy}, $d_{x^2-y^2}/\sigma^*$, $d'_{yz/xz}$, d'_{z^2}, $d'_{yz/xz}$.

Spin-free State	Spin	Weight	CI Vectors (Weights)
16	0.0	0.367	22u222d22000 (0.704)
30	1.0	0.175	22u222u22000 (0.767)
29	1.0	0.173	2u2222u22000 (0.774)
31	1.0	0.143	u22222u22000 (0.850)

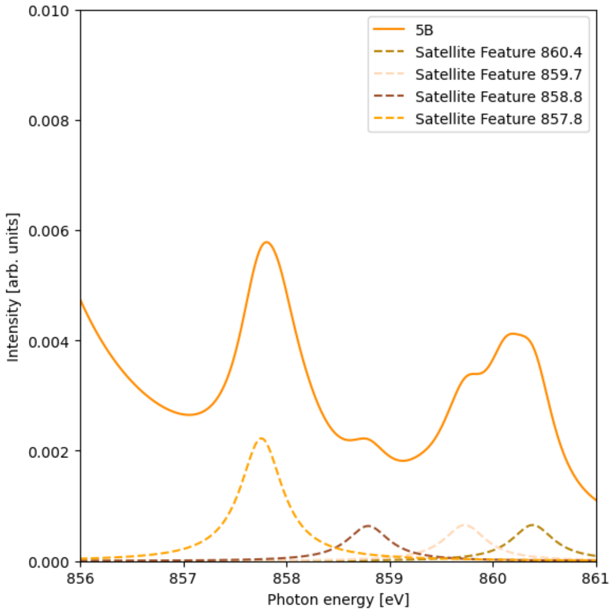

Figure S19: Contributions to the satellite peaks for **5B**. Lorentzian broadened with gamma 0.7.

Complex 1D MLCT

L_3-Edge

Table S41: The spin free states that contribute at least 5% to peak A in 1D. The order of the orbitals in the CI vector is p_x, p_y, p_z, $d_{yz/xz}$, d_{z^2}, $d_{x^2-y^2}/\sigma$, $d_{yz/xz}$, d_{xy}, $d'_{yz/xz}$, d'_{z^2}, $d_{x^2-y^2}/\sigma^*$, π^*.

Spin-free State	Spin	Weight	CI Vectors (Weights)
4	0.0	0.451	u222222200d0 (0.763)<br>u222d2220020 (0.055)<br>u2222d220020 (0.051)
19	1.0	0.214	u222222200u0 (0.641)<br>2u22222200u0 (0.193)
3	0.0	0.132	2u22222200d0 (0.741)<br>2u22d2220020 (0.121)
18	1.0	0.123	22u2222200u0 (0.853)

Table S42: The spin free states that contribute at least 5% to peak B in 1D. The order of the orbitals in the CI vector is p_x, p_y, p_z, $d_{yz/xz}$, d_{z^2}, $d_{x^2-y^2}/\sigma$, $d_{yz/xz}$, d_{xy}, $d'_{yz/xz}$, d'_{z^2}, $d_{x^2-y^2}/\sigma^*$, π^*.

Spin-free State	Spin	Weight	CI Vectors (Weights)
3	0.0	0.435	2u22222200d0 (0.741)<br>2u22d2220020 (0.121)
17	1.0	0.196	2u22222200u0 (0.653)<br>u222222200u0 (0.189)
18	1.0	0.128	22u2222200u0 (0.853)
4	0.0	0.117	u222222200d0 (0.763)<br>u222d2220020 (0.055)<br>u2222d220020 (0.051)

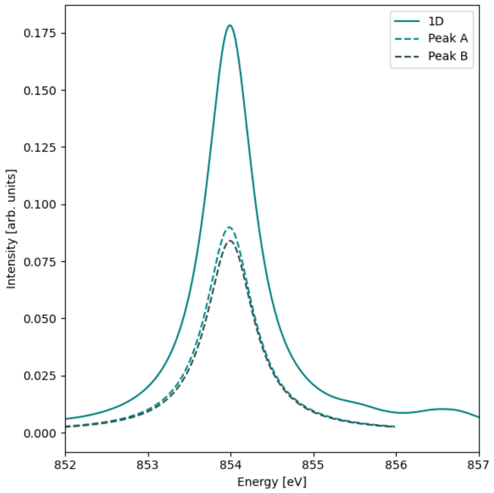

Figure S20: Contributions to the L_3 edge for 1D. Lorentzian broadened with gamma 0.7.

Satellite Peaks

Table S43: The spin free states that contribute at least 10% to the satellite feature at 855.6 eV in 1D. The order of the orbitals in the CI vector is p_x, p_y, p_z, $d_{yz/xz}$, d_{z^2}, $d_{x^2-y^2}/\sigma$, $d_{yz/xz}$, d_{xy}, $d'_{yz/xz}$, d'_{z^2}, $d_{x^2-y^2}/\sigma^*$, π^*.

Spin-free State	Spin	Weight	CI Vectors (Weights)
27	1.0	0.252	22u2222u0020 (0.119)
			2u2222u20020 (0.262)
			u22222u20020 (0.114)
			22u2u2220020 (0.214)
24	1.0	0.212	2u2u22220020 (0.508)
			u22u22220020 (0.123)
25	1.0	0.103	u222222u0020 (0.521)

Table S44: The spin free states that contribute at least 10% to the satellite feature at 856.5 eV in 1D. The order of the orbitals in the CI vector is p_x, p_y, p_z, $d_{yz/xz}$, d_{z^2}, $d_{x^2-y^2}/\sigma$, $d_{yz/xz}$, d_{xy}, $d'_{yz/xz}$, d'_{z^2}, $d_{x^2-y^2}/\sigma^*$, π^*.

Spin-free State	Spin	Weight	CI Vectors (Weights)
30	1.0	0.319	2u2222u20020 (0.154)
			u22222u20020 (0.630)
10	0.0	0.239	2u22222d0020 (0.196)
			u222222200d0 (0.121)
			u222d2220020 (0.499)
			22ud22220020 (0.103)
23	1.0	0.145	22u2222u0020 (0.114)
			22u2u2220020 (0.236)
			22uu22220020 (0.101)
			u22u22220020 (0.227)

Table S45: The spin free states that contribute at least 10% to the satellite feature at 856.8 eV in 1D. The order of the orbitals in the CI vector is p_x, p_y, p_z, $d_{yz/xz}$, d_{z^2}, $d_{x^2-y^2}/\sigma$, $d_{yz/xz}$, d_{xy}, $d'_{yz/xz}$, d'_{z^2}, $d_{x^2-y^2}/\sigma^*$, π^*.

Spin-free State	Spin	Weight	CI Vectors (Weights)
30	1.0	0.297	2u2222u20020 (0.154)
			u22222u20020 (0.630)
11	0.0	0.287	2u22222200d0 (0.138)
			u222222d0020 (0.232)
			2u222d220020 (0.151)
			2u222d220020 (0.306)
			2u22d2220020 (0.107)

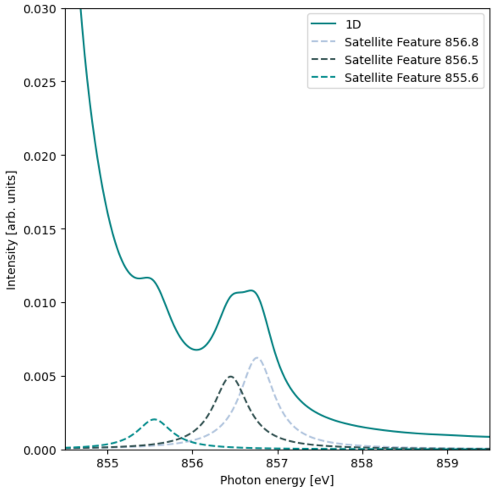

Figure S21: Contributions to the satellite features for 1D. Lorentzian broadened with gamma 0.7.

Complex 1B MLCT

L_3-Edge

Table S46: The spin free states that contribute at least 5% to peak A in **1B**. The order of the orbitals in the CI vector is p_x, p_y, p_z, $d_{yz/xz}$, $d_{yz/xz}$, d_{z^2}, d_{xy}, $d_{x^2-y^2}/\sigma$, $d_{x^2-y^2}/\sigma^*$, π^*, d'_{z^2}, $d'_{yz/xz}$

Spin-free State	Spin	Weight	CI Vectors (Weights)
3	0.0	0.383	2u222222d000 (0.376)<br>u22222220d00 (0.194)<br>22u22222d000 (0.153)<br>2u222d222000 (0.064)
18	1.0	0.287	u2222222u000 (0.894)
4	0.0	0.240	2u222222d000 (0.347)<br>u22222220d00 (0.317)<br>22u22222d000 (0.065)

Table S47: The spin free states that contribute at least 5% to peak B in **1B**. The order of the orbitals in the CI vector is p_x, p_y, p_z, $d_{yz/xz}$, $d_{yz/xz}$, d_{z^2}, d_{xy}, $d_{x^2-y^2}/\sigma$, $d_{x^2-y^2}/\sigma^*$, π^*, d'_{z^2}, $d'_{yz/xz}$

Spin-free State	Spin	Weight	CI Vectors (Weights)
5	0.0	0.343	22u22222d000 (0.557)<br>u22222220d00 (0.178)
19	1.0	0.275	2u222222u000 (0.901)
4	0.0	0.240	2u222222d000 (0.347)<br>u22222220d00 (0.317)<br>22u22222d000 (0.065)
3	0.0	0.082	2u222222d000 (0.376)<br>u22222220d00 (0.194)<br>22u22222d000 (0.153)<br>2u222d222000 (0.064)

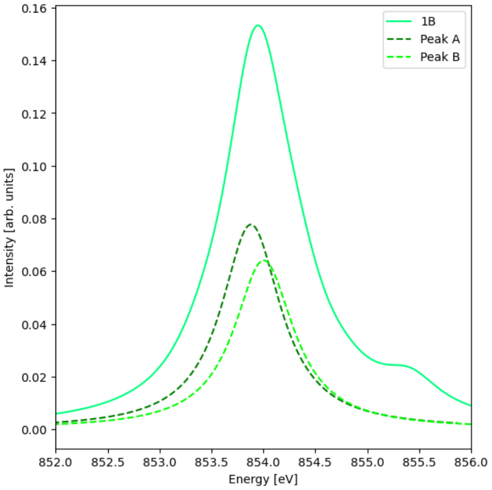

Figure S22: Contributions to the L_3 edge for **1B**. Lorentzian broadened with gamma 0.7.

Satellite Peaks

Table S48: The spin free states that contribute at least 10% to the satellite feature at 857.4 eV in 1B. The order of the orbitals in the CI vector is p_x, p_y, p_z, $d_{yz/xz}$, $d_{yz/xz}$, d_{z^2}, d_{xy}, $d_{x^2-y^2}/\sigma$, $d_{x^2-y^2}/\sigma^*$, π^*, d'_{z^2}, $d'_{yz/xz}$

Spin-free State	Spin	Weight	CI Vectors (Weights)
31	1.0	0.425	22u22u222000 (0.364)
			u222u2222000 (0.280)
			2u2u22222000 (0.228)
9	0.0	0.250	22u22d222000 (0.415)
			2u222d222000 (0.146)
			u222d2222000 (0.107)
28	1.0	0.124	2u22u2222000 (0.809)
27	1.0	0.110	u222u2222000 (0.507)
			2u2u22222000 (0.317)

Table S49: The spin free states that contribute at least 10% to the satellite feature at 858.1 eV in 1B. The order of the orbitals in the CI vector is p_x, p_y, p_z, $d_{yz/xz}$, $d_{yz/xz}$, d_{z^2}, d_{xy}, $d_{x^2-y^2}/\sigma$, $d_{x^2-y^2}/\sigma^*$, π^*, d'_{z^2}, $d'_{yz/xz}$

Spin-free State	Spin	Weight	CI Vectors (Weights)
30	1.0	0.346	2u222u222000 (0.569)
			u2222u222000 (0.120)
			22uu22222000 (0.181)
9	0.0	0.299	22u22d222000 (0.415)
			2u222d222000 (0.146)
			u222d2222000 (0.107)
28	1.0	0.150	2u22u2222000 (0.809)

Table S50: The spin free states that contribute at least 10% the satellite feature at 858.6 eV in 1B. The order of the orbitals in the CI vector is p_x, p_y, p_z, $d_{yz/xz}$, $d_{yz/xz}$, d_{z^2}, d_{xy}, $d_{x^2-y^2}/\sigma$, $d_{x^2-y^2}/\sigma^*$, π^*, d'_{z^2}, $d'_{yz/xz}$

Spin-free State	Spin	Weight	CI Vectors (Weights)
12	0.0	0.287	u22d22222000 (0.102)
			u22u2222dd00 (0.128)
13	0.0	0.212	u22u2222dd00 (0.165)
15	0.0	0.102	u2222u22dd00 (0.241)
			u2222u2d2d00 (0.145)

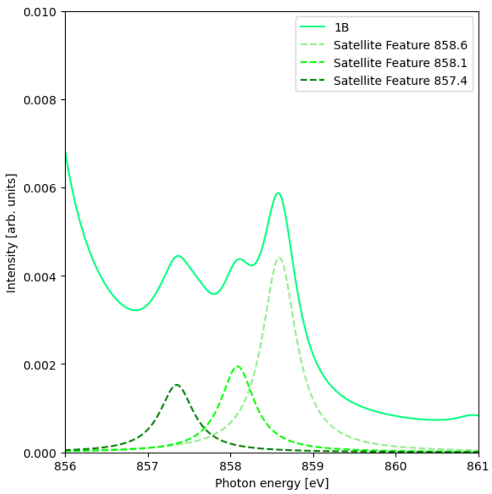

Figure S23: Contributions to the satellite peaks for **1B**. Lorentzian broadened with gamma 0.7.

Complex 5D MLCT

L_3-Edge

Table S51: The spin free states that contribute at least 5% to peak A in 5D. The order of the orbitals in the CI vector is p_x, p_y, p_z, $d_{yz/xz}$, d_{xy}, $d_{yz/xz}$, $d_{x^2-y^2}/\sigma$, d_{z^2}, $d'_{yz/xz}$, π^*, $d_{x^2-y^2}/\sigma^*$, d'_{z^2}.

Spin-free State	Spin	Weight	CI Vectors (Weights)
3	0.0	0.573	2u22222200d0 (0.606)
			2u22222d0020 (0.139)
			22u22d220020 (0.059)
17	1.0	0.222	u222222200u0 (0.751)
			2u22222200u0 (0.135)
18	1.0	0.079	22u2222200u0 (0.896)

Table S52: The spin free states that contribute at least 5% to peak B in 5D. The order of the orbitals in the CI vector is p_x, p_y, p_z, $d_{yz/xz}$, d_{xy}, $d_{yz/xz}$, d_{z^2}, $d_{x^2-y^2}/\sigma$, $d'_{yz/xz}$, π^*, $d_{x^2-y^2}/\sigma^*$, d'_{z^2} .

Spin-free State	Spin	Weight	CI Vectors (Weights)
4	0.0	0.548	u222222200d0 (0.653)
			u222222d0020 (0.122)
			2u22d2220020 (0.073)
19	1.0	0.250	2u22222200u0 (0.744)
			u222222200u0 (0.132)
18	1.0	0.065	22u2222200u0 (0.896)

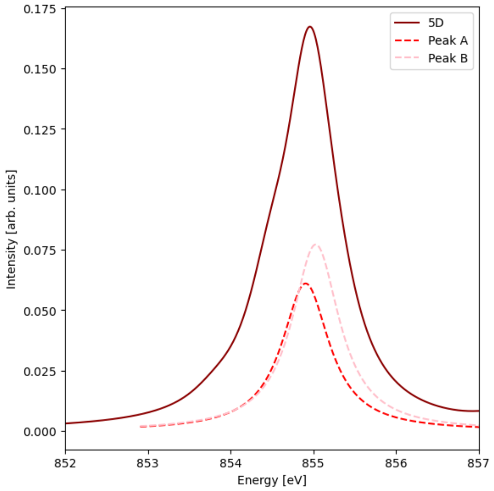

Figure S24: Contributions to the L_3 edge for 5D. Lorentzian broadened with gamma 0.7.

Satellite Peak

Table S53: The spin free states that contribute at least 10% to the satellite feature at 857.0 eV in 5D. The order of the orbitals in the CI vector is p_x, p_y, p_z, $d_{yz/xz}$, d_{z^2}, $d_{x^2-y^2}/\sigma$, $d_{yz/xz}$, d_{xy}, $d'_{yz/xz}$, d'_{z^2}, $d_{x^2-y^2}/\sigma^*$, π^*.

Spin-free State	Spin	Weight	CI Vectors (Weights)
22	1.0	0.495	u2222u220020 (0.224)
			22u2u2220020 (0.462)
11	0.0	0.143	2u22222200d0 (0.238)
			2u22222d0020 (0.140)
			2u2222d20020 (0.141)
			22u22d220020 (0.149)
			u222d2220020 (0.181)
30	1.0	0.137	u222222u0020 (0.160)
			2u22u2220020 (0.362)
			u22u22220020 (0.149)

Table S54: The spin free states that contribute at least 10% to the satellite feature at 857.1 eV in 5D. The order of the orbitals in the CI vector is p_x, p_y, p_z, $d_{yz/xz}$, d_{z^2}, $d_{x^2-y^2}/\sigma$, $d_{yz/xz}$, d_{xy}, $d'_{yz/xz}$, d'_{z^2}, $d_{x^2-y^2}/\sigma^*$, π^*.

Spin-free State	Spin	Weight	CI Vectors (Weights)
29	1.0	0.248	u2222u220020 (0.429)
			22u2u2220020 (0.223)
28	1.0	0.238	2u22222u0020 (0.104)
			22u22u220020 (0.196)
			u222u2220020 (0.380)
			2u2u22220020 (0.127)
10	0.0	0.182	u222222200d0 (0.197)
			u222222d0020 (0.324)
			2u22d2220020 (0.155)
27	1.0	0.139	u222222u0020 (0.136)
			2u222u220020 (0.262)
			u22u22220020 (0.231)
24	1.0	0.123	22u22u220020 (0.394)
			u222u2220020 (0.322)

Table S55: The spin free states that contribute at least 10% to the satellite feature at 857.2 eV in 5D. The order of the orbitals in the CI vector is p_x, p_y, p_z, $d_{yz/xz}$, d_{z^2}, $d_{x^2-y^2}/\sigma$, $d_{yz/xz}$, d_{xy}, $d'_{yz/xz}$, d'_{z^2}, $d_{x^2-y^2}/\sigma^*$, π^*.

Spin-free State	Spin	Weight	CI Vectors (Weights)
27	1.0	0.334	u222222u0020 (0.136)
			2u222u220020 (0.262)
			u22u22220020 (0.231)
29	1.0	0.212	u2222u220020 (0.429)
			22u2u2220020 (0.223)
31	1.0	0.207	2u22222u0020 (0.370)
			2u2u22220020 (0.382)
11	0.0	0.172	2u22222200d0 (0.238)
			2u22222d0020 (0.140)
			2u2222d20020 (0.140)
			22u22d220020 (0.149)
			u222d2220020 (0.181)

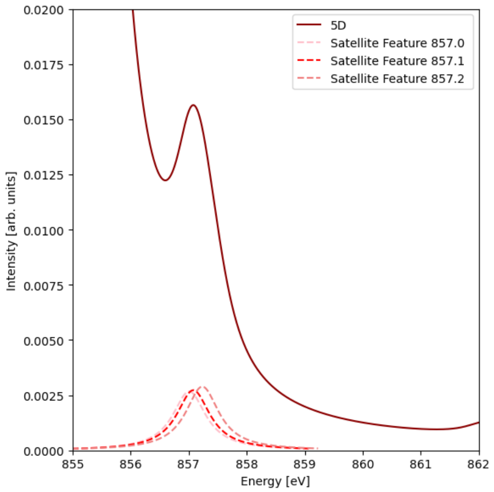

Figure S25: Contributions to the satellite feature for 5D. Lorentzian broadened with gamma 0.7.

Complex 5B MLCT

L_3-Edge

Table S56: The spin free states that contribute at least 5% to peak A in 5B. The order of the orbitals in the CI vector is p_x, p_y, p_z, $d_{yz/xz}$, $d_{yz/xz}$, d_{z^2}, $d_{x^2-y^2}/\sigma$, d_{xy}, $d_{x^2-y^2}/\sigma^*$, $d'_{yz/xz}$, π^*, d'_{z^2}.

Spin-free State	Spin	Weight	CI Vectors (Weights)
3	0.0	0.550	2u222222d000 (0.602)<br>22u22222d000 (0.136)<br>22u22d222000 (0.107)<br>u222d2222000 (0.057)
18	1.0	0.251	2u222222u000 (0.716)<br>22u22222u000 (0.191)
4	0.0	0.087	22u22222d000 (0.607)<br>2u222222d000 (0.150)<br>2u222d222000 (0.116)
17	1.0	0.070	u2222222u000 (0.893)

Table S57: The spin free states that contribute at least 5% to peak B in 5B. The order of the orbitals in the CI vector is p_x, p_y, p_z, $d_{yz/xz}$, $d_{yz/xz}$, d_{z^2}, $d_{x^2-y^2}/\sigma$, d_{xy}, $d_{x^2-y^2}/\sigma^*$, $d'_{yz/xz}$, π^*, d'_{z^2}.

Spin-free State	Spin	Weight	CI Vectors (Weights)
4	0.0	0.542	22u22222d000 (0.542)<br>2u222222d000 (0.150)<br>2u222d222000 (0.116)
19	1.0	0.293	22u22222u000 (0.726)<br>2u222222u000 (0.194)
3	0.0	0.084	2u222222d000 (0.602)<br>22u22222d000 (0.136)<br>22u22d222000 (0.107)<br>u222d2222000 (0.057)

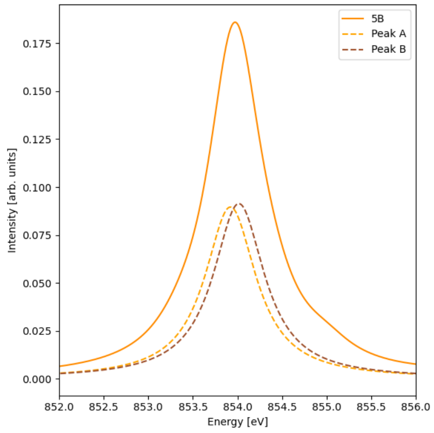

Figure S26: Contributions to the L_3 edge for 5B. Lorentzian broadened with gamma 0.7.

Satellite Peak

Table S58: The spin free states that contribute at least 10% to satellite feature at 856.8 eV in 5B. The order of the orbitals in the CI vector is p_x, p_y, p_z, $d_{yz/xz}$, $d_{yz/xz}$, d_{z^2}, $d_{x^2-y^2}/\sigma$, d_{xy}, $d_{x^2-y^2}/\sigma^*$, $d'_{yz/xz}$, π^*, d'_{z^2}.

Spin-free State	Spin	Weight	CI Vectors (Weights)
9	0.0	0.504	22u22d222000 (0.240)
			2u222d222000 (0.284)
			u22d22222000 (0.143)
31	1.0	0.291	u2222u222000 (0.307)
			22u2u2222000 (0.160)
			2u2u22222000 (0.153)

Table S59: The spin free states that contribute at least 10% to satellite feature at 857.1 eV in 5B. The order of the orbitals in the CI vector is p_x, p_y, p_z, $d_{yz/xz}$, $d_{yz/xz}$, d_{z^2}, $d_{x^2-y^2}/\sigma$, d_{xy}, $d_{x^2-y^2}/\sigma^*$, $d'_{yz/xz}$, π^*, d'_{z^2}.

Spin-free State	Spin	Weight	CI Vectors (Weights)
10	0.0	0.410	22u22222d000 (0.108)
			22u22d222000 (0.137)
			2u222d222000 (0.166)
			u222d2222000 (0.336)
24	1.0	0.172	2u222u222000 (0.303)
			u22u22222000 (0.326)
31	1.0	0.119	u2222u222000 (0.307)
			22u2u2222000 (0.160)
			2u2u22222000 (0.153)

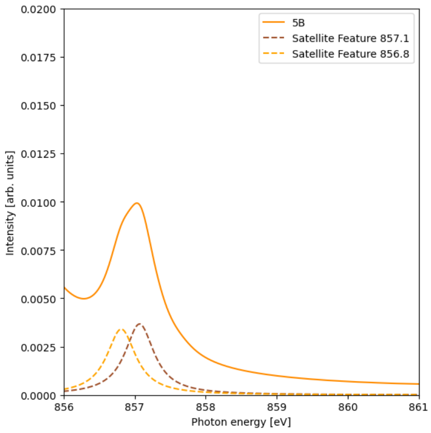

Figure S27: Contributions to the satellite feature for **5B**. Lorentzian broadened with gamma 0.7.

References

(1) Cagan, D. A.; Bím, D.; Silva, B.; Kazmierczak, N. P.; McNicholas, B. J.; Hadt, R. G. Elucidating the mechanism of excited-state bond homolysis in nickel–bipyridine photoredox catalysts. *J. Am. Chem. Soc.* **2022**, *144*, 6516–6531, DOI: https://doi.org/10.1021/jacs.2c01356.

(2) Bao, J. J.; Truhlar, D. G. Automatic active space selection for calculating electronic excitation energies based on high-spin unrestricted hartree–fock orbitals. *J. Chem. Theory Comput.* **2019**, *15*, 5308–5318, DOI: https://doi.org/10.1021/acs.jctc.9b00535.

(3) King, D. S.; Gagliardi, L. A ranked-orbital approach to select active spaces for high-throughput multireference computation. *J. Chem. Theory Comput.* **2021**, *17*, 2817–2831, DOI: https://doi.org/10.1021/acs.jctc.1c00037.

(4) Nelson, K. J.; Kazmierczak, N. P.; Cagan, D. A.; Follmer, A. H.; Scott, T. R.; Raj, S. L.; Garratt, D.; Powers-Riggs, N.; Gaffney, K. J.; Hadt, R. G.; Cordones, A. A. Multiconfigurational Electronic Structure of Nickel Cross-Coupling Catalysts Revealed by X-ray Absorption Spectroscopy. *J. Phys. Chem. Lett.* **2024**, *16*, 87–94, DOI: https://doi.org/10.1021/acs.jpclett.4c02917.
